# A meta‐analysis of sexual orientation inequities in substance use among youth

**DOI:** 10.1111/add.70301

**Published:** 2025-12-24

**Authors:** Ethan H. Mereish, Hyemin Lee, Jeremy T. Goldbach, Sophie Hathaway, Emily A. Hennessy

**Affiliations:** ^1^ Lavender Lab, Department of Psychology University of Maryland College Park MD USA; ^2^ Department of Health and Exercise Science University of Oklahoma Norman OK USA; ^3^ Washington University in St. Louis St. Louis MO USA; ^4^ Harvard University Cambridge MA USA; ^5^ Recovery Research Institute Harvard Medical School Boston MA USA; ^6^ Center for Addiction Medicine Massachusetts General Hospital Boston MA USA

**Keywords:** adolescents, disparities, inequities, meta‐analysis, sexual minority, substance use, youth

## Abstract

**Background and Aims:**

Sexual minority youth report significantly higher rates of substance use than heterosexual youth, yet a comprehensive and systematic evaluation and synthesis of the magnitude of this inequity has not been conducted. The purpose of this study was to conduct a meta‐analysis to assess the magnitude of overall inequities in substance use between sexual minority and heterosexual youth and examine demographic and methodological moderating factors that might impact variability in substance use patterns.

**Methods:**

A meta‐analysis of studies that examined sexual orientation inequities in substance use. We conducted a comprehensive literature search across four electronic databases (PubMed, APA PsycINFO, Web of Science, ProQuest; January 2008–July 2024). Studies were eligible if they included a youth participant population (mean sample age of 25 years or younger), examined differences in substance use between sexual minority and heterosexual groups, and published between 2008 and 2024 in English. Primary outcomes were any measures of substance use; secondary outcome was age of substance use initiation. Following PRISMA guidelines, reviewers independently reviewed and extracted data. Analyses employed random‐effects models, with robust variance estimation to account for dependency among multiple effect sizes within studies. Analyses examined continuous and dichotomous outcomes separately. Bivariate meta‐regression examined moderators.

**Results:**

Among 304 studies of 5 928 282 youth, sexual minority youth reported greater quantity and frequency of all assessed substances (i.e. alcohol, nicotine, cannabis, prescription drugs, powder cocaine, crack cocaine, meth/amphetamine, 3,4‐methylenedioxymethamphetamine, heroin, and other substances) and engaged in more polysubstance use than heterosexual youth. For continuous outcomes, Hedges' g ranged from 0.10 (95% confidence interval [CI], 0.05–0.15, I^2^ = 99.52, participants = 354 201, studies = 48) for alcohol to 0.40 (95% CI, 0.21–0.59, I^2^ = 100.00, participants = 30 679, studies = 5) for mixed/polysubstance use. Dichotomous outcomes showed consistently elevated odds ratios, ranging from 1.34 (95% CI: 1.24–1.46, I^2^ = 96.01, participants = 3 500 203, studies = 128) for alcohol to 4.63 (95% CI, 2.91–7.38, I^2^ = 86.35, participants = 391 827, studies = 10) for heroin use. Sexual minority youth also had earlier ages of initiation (all substance outcomes: odds ratio, 1.45; 95% CI, 1.04–2.03, I^2^ = 95.39, participants = 619 187, studies = 11). Moderation results indicated that inequities were larger for plurisexual youth (e.g. bisexual, pansexual), sexual minority girls and young women, and adolescents 18 years of age or younger. The magnitude of inequities was also larger for lifetime measures of use compared with measures of recent use.

**Conclusions:**

Sexual minority youth — particularly those who are plurisexual, sexual minority girls and young women, and adolescents 18 years of age or younger — appear to engage in substance use at higher rates than heterosexual youth.

## INTRODUCTION

Hazardous substance use among youth (i.e. adolescents and young adults up to 25 years of age) is a serious public health issue that has a myriad of negative effects on health in adolescence and into adulthood [1, 2, 3, 4]. Sexual minority youth (SMY) (i.e. youth who identify as lesbian, gay, bisexual, queer, and/or youth who have romantic/sexual attractions to or engage in romantic/sexual behaviors with the same‐sex or same‐gender and/or multiple sexes or genders) in particular report significantly higher rates of substance use compared to heterosexual youth [5, 6]. SMY use substances at younger ages, use more frequently and at greater quantities and have increased risk for developing substance use disorders (SUDs) than heterosexual youth in adolescence and into adulthood [5, 6, 7, 8, 9, 10, 11, 12, 13, 14, 15]. Disparities in substance use, and particularly hazardous use, are considered inequities as they are rooted in intersecting systems of oppression (e.g. heterosexism, anti‐bisexual stigma, cissexism and racism), and hazardous use is a preventable outcome of socially unjust systems [6]. Consistent with motivational models of substance use and the oppression framework, SMY may engage in substance use for a variety of reasons (e.g. coping, social, enhancement and conformity), and some reflect their coping with and resistance of oppression‐based stressors (e.g. find community, feel liberated), lack of social safety and socially unjust conditions [5, 6, 16].

Substance use prevalence among SMY vary substantially by socio‐demographic and measurement factors [15, 17, 18, 19, 20, 21, 22]. Plurisexual youth (i.e. youth who are attracted to more than one sex or gender; e.g. bisexual and pansexual) and sexual minority girls and young women have the highest rates of substance use [5, 6, 15, 20]. Additionally, there is limited, mixed evidence that indicates that questioning youth (i.e. youth who are questioning or unsure of their sexual orientation) have higher rates of substance use than their heterosexual peers [23, 24, 25]. Moreover, sexual orientation is a multi‐dimensional construct, encompassing identity (e.g. lesbian), attraction (e.g. romantic or sexual attractions to a similar sex or gender or multiple sexes or genders) and/or behavioral (e.g. sexual behaviors with a similar sex or gender or multiple sexes or genders) dimensions [26]. Inequities in substance use vary based on the dimension(s) of sexual orientation measured [21].

Despite well‐documented sexual orientation inequities in substance use among youth, there has been only one systematic review and meta‐analysis of this literature [13]. This study is outdated and limited as it was published in 2008 and only included 18 studies. There has been a significant proliferation of research on sexual orientation inequities over the past 17 years, as these inequities have been acknowledged as federal public health priorities in some countries [e.g. United States (US), Australia], and there has been an increase in the measurement of sexual orientation in research [26, 27, 28]. An updated and precise understanding of sexual orientation inequities in substance use is warranted. Therefore, the purpose of this pre‐registered systematic review and meta‐analysis was to assess the magnitude of overall inequities in substance use and age of initiation between SMY and heterosexual youth, examine the variability in substance use inequities, and identify demographic and methodological factors that might influence this variability.

## METHODS

This meta‐analysis is reported in line with the Preferred Reporting Items for Systematic Reviews and Meta‐analyses (PRISMA) reporting guidelines [29]. The protocol, analysis plan and coding manual were pre‐registered on the Open Science Framework (https://osf.io/9r68z). Although we followed the pre‐registration, additional analyses were conducted in response to the peer review process.

### Data sources and search strategy

We conducted a comprehensive literature search across four electronic databases (PubMed, APA PsycINFO, Web of Science and ProQuest) from January 2008 through July 2024. The search began in 2008 because the last meta‐analysis of sexual orientation disparities among youth was published at that time [13]. Search terms were developed through consultation with a university librarian and based on previous meta‐analyses of substance use among sexual minority populations [13, 16, 20, 30, 31, 32]. Complete database search strategies are provided in the Appendix S1 of the Supporting information.

### Eligibility criteria and screening

Studies were eligible if they met the following inclusion criteria: (1) included a participant sample of youth with a mean age of 25 years or younger; (2) assessed substance use and examined differences in substance use between heterosexual and sexual minority groups (defined broadly as any sexual orientation dimension based on identity, attraction or behaviors); (3) published between January 2008 and July 2024; and (4) written in English. All included studies used observational designs, and no experimental studies met our inclusion criteria. Our search included both peer‐reviewed journal articles and gray literature (i.e. dissertations, theses). If data from the same sample were published in a peer‐reviewed article and dissertation, we retained only the peer‐reviewed article. We excluded duplicates, reviews and manuscripts without accessible full text or relevant effect size information identified. As we did not have the resources to search for gray literature through other types of website searches, conference abstracts, white papers and book chapters were not included. Reviewers independently screened titles and abstracts of all identified records, followed by full‐text reviews of potentially eligible studies.

### Outcome

The primary outcomes of interest encompassed any measure of substance use, including alcohol, nicotine, cannabis, prescription drugs, cocaine, methamphetamine/amphetamine (meth/amphetamine), 3,4‐methylenedioxymethamphetamine (MDMA) (e.g. molly), crack, heroin, other substance use (e.g. inhalants, sedatives and hallucinogens) or mixed/polysubstance use. Quantity measures captured how much substance was used (e.g. number of drinks per day, cigarettes smoked per day) and included binary prevalence measures (any use vs. no use). Frequency measures captured how often substances were used over time (e.g. number of days used per month, percentage of use days over past 30 days). The secondary outcome was age of substance use initiation, which referred to the age at which participants first used a substance.

### Data extraction

Using a standardized coding form, each study was extracted by two independent reviewers. Extracted information included: study (e.g. sampling strategy, inclusion/exclusion criteria), sample demographic (e.g. age, sex, gender, race/ethnicity and sexual orientation), outcome (e.g. type of substance use, duration of use) characteristics and statistical data needed for effect size calculation (e.g. sample sizes, means). Coders were trained by the first author, who is a doctoral‐level researcher with expertise in SMY's substance use, and coders met weekly to discuss the coding process. Discrepancies were resolved through discussion and consensus. We contacted corresponding authors of studies with unreported or partially reported effect size data for eligible outcomes.

### Effect size estimation

For continuous outcomes, we first calculated Cohen's *d* and then transformed them to Hedges' g, which applies small study bias correction, to measure standardized mean differences in substance use between SMY and heterosexual youth. Positive values indicate higher substance use among SMY than heterosexual youth. Effect sizes are interpreted within the context of substance use research rather than using generic interpretation guidelines, with consideration given to the magnitude of differences observed and their practical significance. For dichotomous outcomes, we calculated and synthesized log odds ratios (ORs) (exponentiated after the synthesis for interpretation) to quantify the relative likelihood of substance use between SMY and heterosexual youth (i.e. OR >1 indicates higher likelihood for SMY; OR <1 indicates lower likelihood for SMY). Following established recommendations [33, 34], we winsorized effect sizes that were three or more standard deviations from the mean to minimize the disproportionate influence of extreme outliers on pooled estimates.

### Statistical analysis

Analyses used random‐effects models using the robumeta command (which conducts meta‐analysis with robust variance estimation) for STATA/MP 16.0 [35], with robust variance estimation (RVE) to account for dependency among multiple effect sizes within studies. Effect sizes were calculated and analyzed separately for each substance type (e.g. alcohol, nicotine and cannabis) to allow for substance‐specific comparisons. We analyzed continuous and dichotomous outcomes separately because they require different effect size metrics representing their underlying distributions (Hedges' g vs. log OR). Although transformations are available to put these in the same type of metric (e.g. transform g to OR), this is not appropriate given their different distributional properties. Random effects inverse variance‐weighted mean effect sizes and 95% CI were calculated using an assumed average within‐study correlation between effects of 0.70 based on current RVE guidelines [35]. For samples that appeared in multiple studies, we included the larger sample size when there was complete overlap, while samples with less than 50% overlap were treated as distinct. For the analyses with too few studies/effects for RVE (i.e. when k < 40 studies and on average <5 effect sizes per study) [35], we used the *metan* command (which performs standard meta‐analysis for independent effect sizes). In these cases, rather than averaging within‐study effects, we selected appropriate effect sizes to synthesize across studies and only selected one from each study. 1 This approach ensured statistical independence of effect sizes as required by the *metan* command, while maintaining the distinction between different outcome types (i.e. quantity, frequency and age of initiation). Missing data on effect sizes and subgroups were handled using complete case analysis.

### Moderator analyses

Moderator analyses examined potential effect size variations through bivariate meta‐regression with RVE to identify sources of heterogeneity and populations with greatest disparities. Study‐level moderators examined whether aggregate study characteristics predicted effect size magnitude, including publication year, sampling strategy (probability vs. convenience sampling), publication type (journal article vs. dissertation/thesis), country (United States vs. other countries), mean age (≤18 vs. >18 years), percentage of White participants and college student sample (yes/no). 2 Subgroup analyses examined participant moderators where effect sizes could be calculated separately for participant subgroups within studies, including sex/gender 3 (effect sizes for female vs. male participants), sexual orientation category and sexual orientation measurement type (identity vs. behavior vs. attraction). For sexual orientation category, we combined across identity, attraction and behavior dimensions. This means that regardless of whether sexual orientation was measured via identity, attraction and/or behavior in the original studies, we categorized participants into four groups: (1) studies that aggregated all sexual minority individuals combined into one group; (2) lesbian/gay individuals or those with same‐sex attraction/sexual partners; (3) bisexual/plurisexual individuals or those with multiple‐sex attractions/sexual partners or who identified as mostly heterosexual; and (4) those who were unsure/questioning. This approach allows us to analyze differences between sexual orientation subgroups while accommodating the diverse measurement approaches across studies. Substance‐related moderators examined whether outcome characteristics influenced effect sizes, including outcome measurement type (quantity vs. frequency) and duration (lifetime vs. recent use). Recent use encompassed any past‐year use, including past 12 months, 6 months, 90 days, 30 days, 2 weeks, week or daily use, while lifetime use referred to any use throughout participants' lives. Moderator analyses used per‐analysis missing data exclusion, with only studies missing the relevant moderator variable excluded from each specific analysis, to maximize the available data for each individual moderator test.

### Heterogeneity and reporting biases

Heterogeneity was assessed using τ^2^, I^2^ and the Q‐statistic [36]. The τ^2^ statistic measures the estimated between‐study variance (>0.50 suggesting substantial heterogeneity), I^2^ estimates what proportion of the variation in observed effects is because of variation in true effects rather than chance [37] and the Q‐statistic tests whether observed differences exceed sampling error (significant *P*‐values indicate heterogeneity). Additionally, prediction intervals were calculated to estimate the likely relationships examined in this set of studies in future studies [38]. Publication bias was evaluated through a meta‐regression of effect sizes on standard errors using RVE [39] to account for effect size dependencies (Table S1) and visually examined through contour‐enhanced funnel plots (Figures S2–S3) [40], which should be interpreted cautiously given the dependent effect size structure of our data.

## RESULTS

### Study characteristics

**FIGURE 1:**
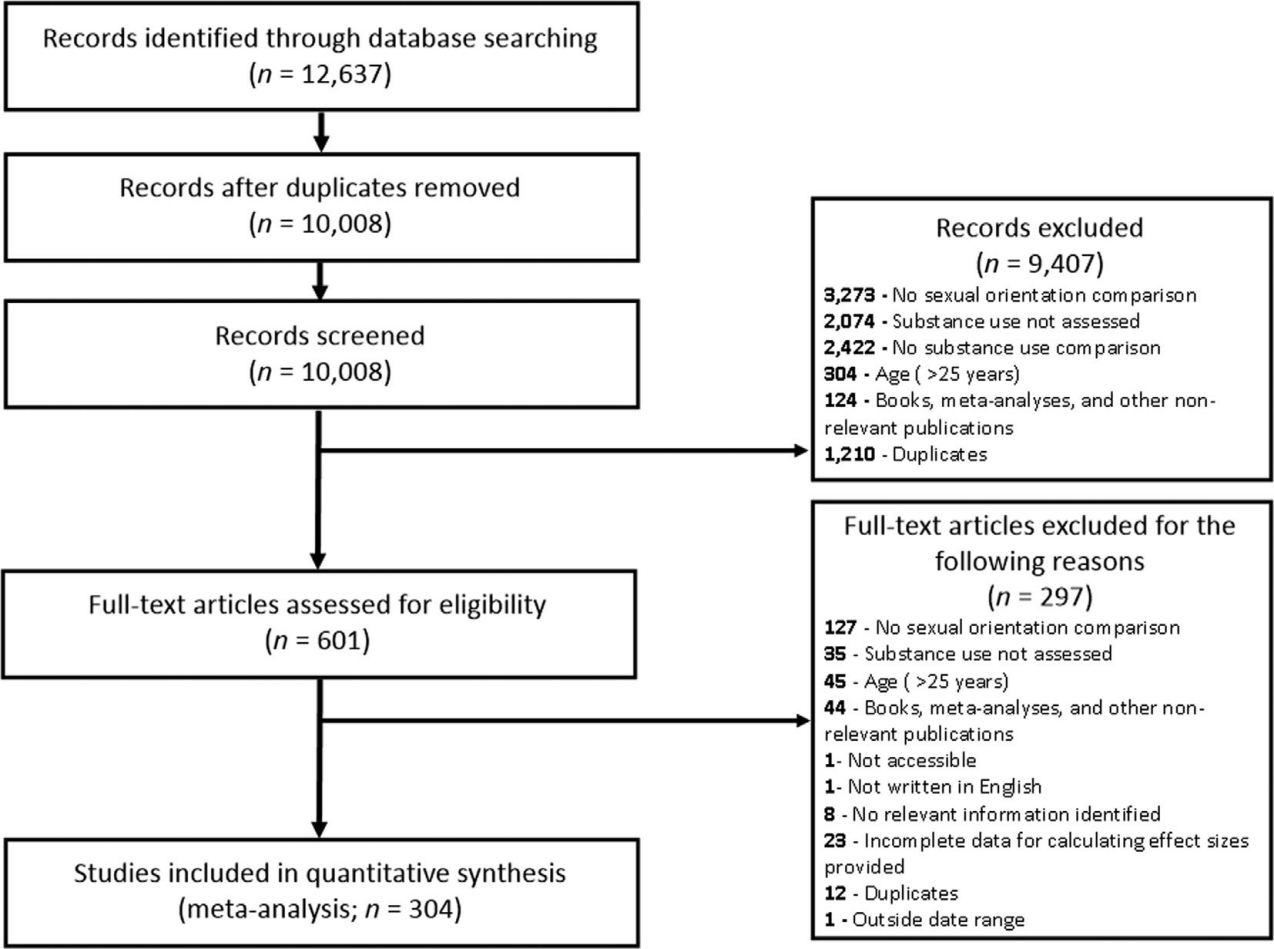
Preferred reporting items for systematic review and meta‐analyses flow diagram.

Among 304 studies (5 928 282 participants) meeting the inclusion criteria, 72 reported continuous and 260 reported dichotomous outcomes, with 28 reporting both types of outcomes (Figure 1; Table 1; Appendix S2 and Appendix S3). The mean participant age was 19.61 years old for continuous outcomes and 19.19 years for dichotomous outcomes, with approximately 36% to 43% under 18 years. Among sexual orientation dimensions, the combined sexual minority group represented 58.9% of continuous and 29.7% of dichotomous outcome studies. Sexual orientation was primarily assessed with identity measures. For substance use outcomes, alcohol was most frequently reported in continuous outcomes, although nicotine was most frequently reported in dichotomous outcomes. Few studies examined age of initiation. Recent use patterns were more commonly assessed than lifetime use history. The number of studies published per year has increased dramatically from 2008 to 2024 (Figure S1).

**TABLE 1:** Summary characteristics of included studies.

Study characteristics	Continuous outcome (*n* = 392, k = 72)			Dichotomous outcome (*n* = 4433, k = 260)		
	*N* (%)/mean (SD)	Range	Missingness	*N* (%)/mean (SD)	Range	Missingness
Study characteristics						
Publication y	2017.61 (5.18)	2008–2024	*n* = 0	2018.21 (3.63)	2008–2024	*n* = 0
Study sampling strategy			*n* = 0			*n* = 0
Convenience sampling	227 (57.9)			1629 (36.8)		
Probability sampling	165 (42.1)			2804 (63.3)		
Publication type			*n* = 0			*n* = 0
Journal article	346 (88.3)			4281 (96.6)		
Dissertation/thesis	46 (11.7)			152 (3.4)		
Country			*n* = 0			*n* = 0
United States	366 (93.4)			3597 (81.1)		
Other countries	26 (6.6)			836 (18.9)		
Mean age (y)		15.39–24.60	*n* = 208		11.50–25.00	*n* = 2662
Continuous variable	19.61 (3.08)			19.19 (3.29)		
18 y or younger (vs. older than 18 y)	67 (36.4)			788 (43.1)		
Transgender identity			*n* = 346			*n* = 3815
% Transgender	0.03 (0.03)	0.00–0.13		0.04 (0.05)	0.00–0.21	
Race/ethnicity			*n* = 32			*n* = 1206
% White, non‐Hispanic/Latine	0.59 (0.18)	0.00–0.93		0.56 (0.21)	0.00–0.95	
College student			*n* = 0			*n* = 49
Yes	111 (28.3)			727 (16.6)		
Participant characteristics						
Sex/gender			*n* = 244			*n* = 1584
Effect sizes for female	104 (70.3)			1517 (53.3)		
Effect sizes for male	44 (29.7)			1332 (46.8)		
Sexual orientation category			*n* = 0			*n* = 37
All sexual minority participants combined into one group	231 (58.9)			1307 (29.7)		
Lesbian/gay, participants with same‐sex attraction/sexual partners	56 (14.3)			1176 (26.8)		
Bisexual/plurisexual, participants with both‐sex attractions/sexual partners/mostly heterosexual	81 (20.7)			1315 (29.9)		
Unsure/questioning	24 (6.1)			598 (13.6)		
Sexual orientation measurement type			*n* = 0			*n* = 0
Identity	357 (91.1)			4054 (91.5)		
Attraction	15 (3.8)			194 (4.4)		
Behavior	14 (3.6)			98 (2.2)		
Mixed	6 (1.5)			87 (2.0)		
Substance outcomes			*n* = 0			*n* = 3
Alcohol	174 (44.4)		*n* = 6	1147 (25.9)		*n* = 92
Any alcohol use	107 (63.7)			648 (61.4)		
Hazardous alcohol use^a^	61 (36.3)			407 (38.6)		
Nicotine	89 (22.7)			1290 (29.1)		
Cannabis	68 (17.4)			550 (12.4)		
Prescription drugs	13 (3.3)			286 (6.5)		
Cocaine	2 (0.5)			110 (2.5)		
Meth/amphetamines	2 (0.5)			144 (3.3)		
Ecstasy/MDMA/molly	0 (0.0)			172 (3.9)		
Crack	1 (0.3)			2 (0.1)		
Heroin	1 (0.3)			77 (1.7)		
Other specific substance use^b^	26 (6.6)			546 (12.3)		
Mixed/poly substance use^c^	16 (4.1)			106 (2.4)		
Substance characteristics						
Outcome measurement type			*n* = 9			*n* = 0
Frequency	205 (53.5)			1889 (42.6)		
Quantity	148 (38.6)			2422 (54.6)		
Age of initiation	30 (7.8)			122 (2.8)		
Outcome measurement duration			*n* = 51			*n* = 316
Lifetime use	33 (9.7)			1469 (36.3)		
Recent use	308 (90.3)		*n* = 0	2621 (63.7)		*n* = 0
Past 12 months/6 months/90 days	107 (31.4)			742 (28.3)		
Past 30 days/2 weeks/week, daily	201 (58.9)			1879 (71.7)		

Abbreviations: k, number of studies; MDMA, 3,4‐methylenedioxymethamphetamine; *n*, number of estimates.

^a^ Hazardous alcohol use includes binge drinking, heavy episodic drinking, alcohol abuse, alcohol intoxication, alcohol use disorder diagnosis and related behaviors.

^b^ Other specific substances include inhalants, sedatives, hallucinogens and other substances not categorized elsewhere.

^c^ Mixed/poly substance use refers to studies reporting use of multiple drug types.

^d^ Recent use encompasses past 12 months, past 6 months, past 90 days, past 30 days, past 2 weeks, past week or daily use.

### Reporting biases

Visual inspection of contour‐enhanced funnel plots conducted separately for continuous (Figure S2) and dichotomous outcomes (Figure S3) revealed some asymmetry, particularly for studies with larger standard errors, suggesting possible small‐study effects. RVE meta‐regressions of effect sizes on standard errors for continuous and dichotomous outcomes showed statistically significant relationships, indicating potential small‐study effects (Table S1).

### Meta‐analysis results by substance outcomes

SMY had significantly elevated rates of substance use compared to heterosexual youth across all substance outcomes (Table 2). For continuous outcomes, Hedges' g ranged from 0.10 (95% CI = 0.05–0.15, I^2^ = 99.52, participants = 354 201, studies = 48) for alcohol to 0.40 (95% CI = 0.21–0.59, I^2^ = 100.00, participants = 30 679, studies = 5) for mixed/polysubstance use. To provide concrete clinical interpretation for the significant alcohol use findings, a supplementary analysis of studies measuring frequency as days per month (studies = 10, effect sizes = 41) estimated that SMY use alcohol approximately 0.36 more days per month compared to heterosexual youth. Dichotomous outcomes showed consistently elevated ORs, ranging from 1.34 (95% CI = 1.24–1.46, I^2^ = 96.01, participants = 3 500 203, studies = 128) for alcohol to 4.63 (95% CI = 2.91–7.38, I^2^ = 86.35, participants = 391 827, studies = 10) for heroin use, indicating SMY had 34% to 363% higher odds of substance use compared to heterosexual peers. Between‐study heterogeneity was high, with Q‐statistics indicating significant variation in both outcome types.

**TABLE 2:** Overall meta‐analysis results by substance outcomes.

Substance outcome types	*n* (k)	Participants *N*	Hedges' g	SE	95% CI	95% PI	*P*‐value	τ^2^	I^2^ (%)	Q‐statistic	Q‐statistic (*P*‐value)
Continuous outcome											
Outcome type: quantity and frequency											
All substance outcomes	360 (70)	551 512	0.18	0.03	0.12–0.24	−0.79‐1.15	<0.001	0.24	99.97	210 000	<0.001
Alcohol	168 (48)	354 201	0.10	0.03	0.05–0.15	−0.26‐0.46	<0.001	0.03	99.52	9406.70	<0.001
Any alcohol use	107 (34)	299 918	0.10	0.03	0.04–0.16	−0.14‐0.33	0.003	0.01	98.54	2073.01	<0.001
Hazardous alcohol use^a^	61 (24)	202 770	0.10	0.04	0.01–0.18	−0.34‐0.54	0.02	0.04	99.70	7423.35	<0.001
Nicotine	82 (22)	251 767	0.19	0.03	0.12–0.26	−0.11‐0.48	<0.001	0.02	94.39	350.93	<0.001
Cannabis	57 (22)	79 678	0.21	0.05	0.10–0.32	−0.53‐0.95	<0.001	0.12	99.50	4236.27	<0.001
Other specific substance use^b^	26 (12)	46 332	0.32	0.08	0.15–0.49	−0.70‐1.34	0.002	0.21	99.99	77035.19	<0.001
Mixed/polysubstance use^c *^	5 (5)	30 679	0.40	0.10	0.21–0.59	−0.22‐1.02	<0.001	0.04	100.00	424978.01	<0.001
Outcome type: age of initiation											
Cannabis initiation*	3 (3)	350 114	−0.31	0.20	−0.70‐0.08	−2.02‐1.40	0.119	0.12	99.97	5796.06	<0.001

Substance outcome types	*n* (k)	Participants *N*	Odds Ratio	SE	95% CI	95% PI	*P*‐value	τ^2^	I^2^ (%)	Q‐statistic	Q‐statistic (*P*‐value)
Dichotomous outcome											
Outcome type: quantity and frequency											
All substance outcomes	4311 (232)	5 675 993	1.60	0.03	1.51–1.70	0.62–4.15	<0.001	0.23	95.68	4750.98	<0.001
Alcohol	1055 (128)	3 500 203	1.34	0.04	1.24–1.46	0.47–3.87	<0.001	0.28	96.01	2947.76	<0.001
Any alcohol use	648 (100)	2 978 433	1.34	0.05	1.22–1.47	0.46–3.93	<0.001	0.29	97.19	3265.03	<0.001
Hazardous alcohol use^a^	407 (77)	2 203 102	1.34	0.06	1.20–1.51	0.41–4.39	<0.001	0.35	97.04	2430.55	<0.001
Nicotine	1265 (133)	3 537 416	1.59	0.04	1.46–1.74	0.60–4.23	<0.001	0.24	94.78	2278.46	<0.001
Cannabis	545 (98)	2 412 216	1.71	0.05	1.56–1.87	0.63–4.64	<0.001	0.25	97.07	3100.13	<0.001
Prescription drugs	286 (34)	497 765	1.72	0.09	1.43–2.06	0.64–4.59	<0.001	0.22	91.10	323.25	<0.001
Cocaine	110 (18)	536 639	2.13	0.13	1.61–2.81	0.75–6.00	<0.001	0.22	89.99	150.16	<0.001
Meth/amphetamines	144 (20)	434 933	2.29	0.17	1.58–3.31	0.44–11.79	<0.001	0.57	84.98	110.80	<0.001
Ecstasy/MDMA/molly	172 (15)	427 034	2.24	0.14	1.65–3.05	0.69–7.22	<0.001	0.26	77.84	50.36	<0.001
Heroin	77 (10)	391 827	4.63	0.20	2.91–7.38	0.97–22.07	<0.001	0.39	86.35	48.66	<0.001
Other specific substance use^b^	546 (82)	1 247 967	2.28	0.06	2.01–2.59	0.65–8.00	<0.001	0.39	96.34	1947.31	<0.001
Mixed/polysubstance use^c^	106 (24)	164 870	1.80	0.10	1.46–2.22	0.20–16.18	<0.001	1.11	95.79	530.42	<0.001
Outcome type: age of initiation											
All substance outcomes	122 (11)	619 187	1.45	0.15	1.04–2.03	0.37–5.72	0.03	0.36	95.39	213.92	<0.001

*Note*: We calculated Hedges' g to measure standardized mean differences with small study bias correction in substance use between SMY and heterosexual youth.

Abbreviations: k, number of studies; MDMA, 3,4‐methylenedioxymethamphetamine; *n*, number of estimates; PI, prediction intervals; SMY, sexual minority youth.

^a^ Hazardous alcohol use includes binge drinking, heavy episodic drinking, alcohol abuse, alcohol intoxication, alcohol use disorder diagnosis and related behaviors.

^b^ Other specific substances include inhalants, sedatives, hallucinogens and other substances not categorized elsewhere.

^c^ Mixed/poly substance use refers to studies reporting use of multiple drug types.

* Analyses were conducted using only one effect size per study via Stata's *metan* syntax rather than robumeta because of small sample sizes (i.e. k < 40 studies and/or <5 effect sizes per study). For mixed/polysubstance use, when studies reported multiple effect sizes, one was selected per study based on: (1) largest sample size to maximize precision; and (2) age most similar to other studies for one longitudinal study with equal sample sizes at multiple time points. For age of initiation for continuous outcomes, only cannabis initiation could be synthesized (k = 3). Additional individual effect sizes for other substance initiation were calculated, but not pooled because of conceptual heterogeneity: Kecojevic *et al*. (2012): opioid (g = −0.18, SE = 0.04), tranquilizer (g = −0.28, SE = 0.04), stimulant (g = −0.18, SE = 0.04), cocaine (g = −0.08, SE = 0.04), methamphetamine (g = 0.03, SE = 0.04), heroin (g = 0.04, SE = 0.04) and crack (g = 0.00, SE = 0.04) initiation; Talley et al. (2019): alcohol (g = −1.92, SE = 0.14) and tobacco (g = −1.92, SE = 0.14) initiation.

Age of initiation analyses for dichotomous outcomes demonstrated that SMY initiated substance use at younger ages than heterosexual youth (all substance outcomes: OR = 1.45; 95% CI = 1.04–2.03, I^2^ = 95.39, participants = 619 187, studies = 11, respectively), indicating SMY had 45% higher odds of earlier substance use initiation compared to heterosexual peers, and studies demonstrated high heterogeneity.

Given that the age inclusion criterion for this review was studies with samples with a mean age of 25 years or younger, there were some studies that had wider age ranges. As such, we also conducted sensitivity analyses excluding studies with upper age ranges in the 30s and older to ensure the findings were specific to studies with youth samples. We found that patterns of results remained consistent for substance use outcomes, age of initiation and the below moderation findings.

### Moderator analyses results

Analyses revealed significant moderating factors across both continuous and dichotomous outcomes. For continuous outcomes (Table 3), significant moderating effects were observed across study participant‐level characteristics (publication year, age and sexual orientation) and measurement‐specific characteristics (duration). Modest temporal changes in substance use inequities were observed over publication years, with disparities decreasing by −0.11 per decade (g = −0.11; 95% CI = −0.22 to −0.01, I^2^ = 99.95, participants = 551 512, studies = 70). Participants 18 years or younger revealed significantly larger disparities compared to those with older participants (g= 0.21; 95% CI = 0.03–0.40, I^2^ = 99.98, participants = 315 647, studies = 44). Identity‐based sexual orientation measurement revealed consistent patterns across subgroups. Specifically, lesbian/gay (g = 0.17; 95% CI = 0.05–0.29, I^2^ = 93.22, participants = 282 868, studies = 17) and bisexual/plurisexual (g = 0.19; 95% CI = 0.10–0.28, I^2^ = 93.78, participants = 285 932, studies = 19) participants showed higher substance use compared to heterosexual participants. Last, greater disparities were observed for lifetime substance use than recent use (g = 0.18; 95% CI = 0.02–0.34, I^2^ = 99.81, participants = 421 642, studies = 59).

**TABLE 3:** Moderator analyses results for continuous outcomes (*n* = 392, k = 72).

Moderator variable	*n* (k)	Participants *N*	Hedges' g	SE	95% CI		*P*‐value	τ^2^	I^2^ (%)	Q‐statistic	Q‐statistic (*P*‐value)
Study characteristics											
Publication y (per decade)	360 (70)	551 512	−0.11	0.05	−0.22	−0.01	0.04	0.09	99.95	62306.04	<0.001
Study sampling strategy											
Probability (vs. convenience)	360 (70)	551 512	0.13	0.07	−0.01	0.28	0.06	0.13	99.96	98294.68	<0.001
Publication type											
Journal article (vs. dissertation/thesis)	360 (70)	551 512	−0.06	0.07	−0.23	0.11	0.41	0.34	100.00	210 000	<0.001
Country											
US (vs. non‐US)	360 (70)	551 512	−0.08	0.05	−0.19	0.03	0.14	0.22	99.99	190 000	<0.001
Mean age (y)											
18 y or younger (vs. older than 18 y)	178 (44)	315 647	0.21	0.09	0.03	0.40	0.03	0.18	99.98	110 000	<0.001
Race/ethnicity											
% White, non‐Hispanic/Latine	328 (61)	393 469	−0.04	0.17	−0.39	0.30	0.80	0.19	99.99	140 000	<0.001
College student											
Yes (vs. no)	360 (70)	551 512	−0.03	0.06	−0.15	0.10	0.69	0.20	99.96	150 000	<0.001
Sample participant characteristics											
Sex/gender											
Effect sizes for female (vs. male)	124 (29)	154 566	−0.14	0.13	−0.42	0.14	0.32	0.12	99.86	16373.57	<0.001
Sexual orientation category (ref: studies that aggregated all sexual minority participants combined into one group)	360 (70)	551 512						0.24	99.99	210 000	<0.001
Lesbian/gay, participants with same‐sex attraction/sexual partners			0.05	0.08	−0.12	0.22	0.53				
Bisexual/plurisexual, participants with both‐sex attractions/sexual partners/mostly heterosexual			0.03	0.06	−0.09	0.15	0.60				
Unsure/questioning			0.45	0.18	0.01	0.88	0.05				
Sexual orientation category (ref: lesbian/gay, participants with same‐sex attraction/sexual partners)	360 (70)	551 512						0.24	99.99	210 000	<0.001
Studies that aggregated all sexual minority participants combined into one group			−0.05	0.08	−0.22	0.12	0.53				
Bisexual/plurisexual, participants with both‐sex attractions/sexual partners/mostly heterosexual			−0.02	0.08	−0.18	0.14	0.81				
Unsure/questioning			0.40	0.18	−0.02	0.81	0.06				
Sexual orientation measurement type (only among lesbian/gay, participants with same‐sex attractions/sexual partners)											
Identity	47 (17)	282 868	0.17	0.06	0.05	0.29	0.008	0.03	93.22	215.35	<0.001
Sexual orientation measurement type (only among bisexual/plurisexual, participants with both‐sex attractions/sexual partners/mostly heterosexual)											
Identity	71 (19)	285 932	0.19	0.04	0.10	0.28	<0.001	0.02	93.78	265.03	<0.001
Substance characteristics											
Outcome measurement type											
Quantity (vs. frequency)	351 (70)	551 512	0.04	0.06	−0.07	0.16	0.45	0.26	99.96	180 000	<0.001
Outcome measurement duration											
Lifetime (vs. recent^a^)	327 (59)	421 642	0.16	0.07	0.00	0.32	0.05	0.02	99.84	4692.31	<0.001
Outcome measurement duration (ref: past 30 days/2 weeks/week, daily)	327 (59)	421 642						0.02	99.81	4692.24	<0.001
Lifetime			0.18	0.07	0.02	0.34	0.04				
Past 12 months/6 months/90 days			0.05	0.06	−0.07	0.16	0.40				

*Note*: We calculated Hedges' g to measure standardized mean differences with small study bias correction in substance use between SMY and heterosexual youth. This table display only studies reporting quantity and frequency outcomes for substance use. Studies measuring other outcome types (i.e. age of initiation) and the transgender identity moderator analysis were excluded because of small sample sizes.

Abbreviations: k, number of studies; *n*, number of estimates; SMY, sexual minority youth; US, United States.

^a^ Recent use encompasses past 12 months, past 6 months, past 90 days, past 30 days, past 2 weeks, past week or daily use.

For dichotomous outcomes (Table 4), significant moderating factors were age, sex and sexual orientation and substance measurement duration. Specifically, participants 18 years old or younger showed 25% higher odds of disparities compared to participants above 18 years (OR = 1.25; 95% CI = 1.05–1.48, I^2^ = 95.80, participants = 1 796 870, studies = 135), indicating that sexual orientation inequities in substance use are particularly pronounced during adolescence. For studies where sex/gender‐specific effect sizes could be calculated, female/women participants demonstrated 21% higher odds of disparities compared to male/men participants (OR = 1.21; 95% CI = 1.05–1.40, I^2^ = 95.09, participants = 2 471 116, studies = 101). When examining sexual orientation categories (I^2^ = 97.54, participants = 5 675 993, studies = 232), aggregated across identity, attraction and behavior, bisexual/plurisexual participants had a greater magnitude in disparities with 28% higher odds (OR = 1.28; 95% CI = 1.16–1.42) compared to lesbian/gay participants. Studies examining unsure/questioning participants showed smaller effect sizes with 31% lower odds compared to studies that combined sexual minority participants into one group (OR = 0.69; 95% CI = 0.58–0.83) and 18% lower odds compared to lesbian/gay participants (OR = 0.82; 95% CI = 0.70–0.96). The analysis of sexual orientation measurement types revealed that among lesbian/gay participants, identity‐based measures showed 45% higher odds (OR = 1.45; 95% CI = 1.30–1.62, I^2^ = 94.53, participants = 2 386 871, studies = 81) and attraction‐based measures showed 68% higher odds (OR = 1.68; 95% CI = 1.14–2.49, I^2^ = 82.17, participants = 43 440, studies = 7) of substance use compared to their heterosexual counterparts, while behavior‐based measures did not reach statistical significance (OR = 1.26; 95% CI = 0.89–1.78, I^2^ = 87.03, participants = 523 110, studies = 10). For bisexual/plurisexual participants, identity‐ and behavior‐based measures also revealed consistent disparities between bisexual/plurisexual participants compared to heterosexual participants, and attraction was not reported as there were too few reports. For bisexual/pansexual participants, effect sizes were larger when sexual orientation was assessed on a behavioral dimension with 139% higher odds (OR = 2.39; 95% CI = 1.74–3.28, I^2^ = 91.41, participants = 520 710, studies = 10) versus an identity dimension with 78% higher odds (OR = 1.78; 95% CI = 1.65–1.92, I^2^ = 94.82, participants = 2 906 772, studies = 89) of substance use than heterosexual participants. Studies examining lifetime substance use showed 14% higher odds of disparities than those examining recent use (OR = 1.14; 95% CI = 1.01–1.29, I^2^ = 97.30, participants = 5 185 079, studies = 221), suggesting that sexual orientation inequities are more pronounced when considering cumulative lifetime experiences rather than current use patterns. Most study‐level characteristics including sampling strategy, publication type, country, race/ethnicity composition, college student status and outcome measurement type (quantity vs. frequency) did not significantly moderate effect sizes across outcome types.

**TABLE 4:** Moderator analyses results for dichotomous outcomes (*n* = 4433, k = 260).

Moderator variable	*n* (k)	Participants *N*	OR	SE	95% CI		*P*‐value	τ^2^	I^2^ (%)	Q‐statistic	Q‐statistic (*P*‐value)
Study characteristics											
Publication y (per decade)	4311 (232)	5 675 993	0.87	0.07	0.75	1.01	0.06	0.25	98.20	4732.93	<0.001
Study sampling strategy											
Probability (vs. convenience)	4311 (232)	5 675 993	1.09	0.06	0.97	1.22	0.15	0.26	95.62	4709.76	<0.001
Publication type											
Journal article (vs. dissertation/thesis)	4311 (232)	5 675 993	0.96	0.11	0.76	1.23	0.74	0.24	99.72	4750.63	<0.001
Country											
US (vs. non‐US)	4311 (232)	5 675 993	0.91	0.08	0.77	1.08	0.27	0.23	98.90	4652.51	<0.001
Mean age (y)											
18 y or below (vs. above 18 y)	1776 (135)	1 796 870	1.25	0.09	1.05	1.48	0.01	0.25	95.80	2404.71	<0.001
Transgender identity											
% Transgender	612 (56)	1 683 823	0.16	0.93	0.02	1.55	0.10	0.22	99.71	2110.71	<0.001
Race/ethnicity											
% White, non‐Hispanic/Latine	3135 (182)	5 008 660	0.76	0.15	0.56	1.03	0.08	0.21	98.06	3591.27	<0.001
College student											
Yes (vs. no)	4262 (231)	5 672 198	0.92	0.07	0.80	1.06	0.25	0.26	98.75	4746.15	<0.001
Participant characteristics											
Sex											
Effect sizes for female (vs. male)	2760 (101)	2 471 116	1.21	0.07	1.05	1.40	0.01	0.42	95.09	1772.70	<0.001
Sexual orientation category (ref: studies that aggregated all sexual minority participants combined into one group)	4274 (232)	5 675 993						0.23	97.54	4541.20	<0.001
Lesbian/gay, participants with same‐sex attraction/sexual partners			0.85	0.06	0.75	0.96	0.01				
Bisexual/plurisexual, participants with both‐sex attractions/sexual partners/mostly heterosexual			1.09	0.06	0.97	1.21	0.14				
Unsure/questioning			0.69	0.09	0.58	0.83	<0.001				
Sexual orientation category (ref: lesbian/gay, participants with same‐sex attraction/sexual partners)	4274 (232)	5 675 993						0.23	97.54	4541.20	<0.001
Studies that aggregated all sexual minority participants combined into one group			1.18	0.06	1.04	1.34	0.01				
Bisexual/plurisexual, participants with both‐sex attractions/sexual partners/mostly heterosexual			1.28	0.05	1.16	1.42	<0.001				
Unsure/questioning			0.82	0.08	0.70	0.96	0.01				
Sexual orientation measurement type (only among lesbian/gay, participants with same‐sex attractions/sexual partners)											
Identity	1013 (81)	2 386 871	1.45	0.06	1.30	1.62	<0.001	0.37	94.53	1258.59	<0.001
Behavior	60 (10)	523 110	1.26	0.15	0.89	1.78	0.16	0.25	87.03	63.40	<0.001
Attraction	37 (7)	43 440	1.68	0.16	1.14	2.49	0.02	0.17	82.17	29.94	<0.001
Sexual orientation measurement type (only among bisexual/plurisexual, participants with both‐sex attractions/sexual partners/mostly heterosexual)											
Identity	1170 (89)	2 906 772	1.78	0.04	1.65	1.92	<0.001	0.24	94.82	1520.34	<0.001
Behavior	50 (10)	520 710	2.39	0.14	1.74	3.28	<0.001	0.19	91.41	84.75	<0.001
Substance characteristics											
Outcome measurement type											
Quantity (vs. frequency)	4311 (232)	5 675 993	1.00	0.06	0.89	1.12	0.99	0.24	97.00	4620.53	<0.001
Outcome measurement duration											
Lifetime (vs. recent^a^)	4051 (221)	5 185 079	1.11	0.06	0.99	1.25	0.08	0.24	97.40	5340.46	<0.001
Outcome measurement duration (ref: past 30 days/2 weeks/week, daily)	4051 (221)	5 185 079						0.25	97.30	5336.68	<0.001
Lifetime			1.14	0.06	1.01	1.29	0.03				
Past 12 months/6 months/90 days			1.09	0.06	0.96	1.24	0.16				

*Note*: This table display only studies reporting quantity and frequency outcomes for substance use. Studies measuring other outcome types (i.e. age of initiation) were excluded because of small sample sizes.

Abbreviations: k, number of studies; *n*, number of estimates; US, United States.

^a^ Recent use encompasses past 12 months, past 6 months, past 90 days, past 30 days, past 2 weeks, past week or daily use.

## DISCUSSION

In this systematic review and meta‐analysis of 304 studies, including 5 928 282 youth, the findings demonstrated that SMY initiated substance use at younger ages, were more likely to use all substances (e.g. alcohol, nicotine, cannabis, opioids, stimulants and other drugs) at a greater frequency and quantity and engaged in more mixed and polysubstance and hazardous substance use than heterosexual youth. These inequities were accentuated based on developmental, demographic and methodological factors. Our findings are consistent with the only previous meta‐analysis of sexual orientation disparities in substance among youth from 2008 [13], and provide an updated, comprehensive and rigorous estimation of sexual orientation inequities in substance use among youth. The magnitude of inequities documented in our study ranged widely, with more accessible and common substances (e.g. alcohol, nicotine) demonstrating smaller effects and illicit drugs (e.g. heroin) having the largest effects. Although the prior meta‐analysis found larger effects across all substances compared to our study, it similarly documented the largest associations for illicit drugs [13]. Our study analyzed inequities separately for continuous and dichotomous outcomes, with more documented significant findings among dichotomous outcomes, likely because there were substantially more reports with dichotomous outcomes, providing more statistical power to detect effects.

Our study's results highlight key demographic differences in substance use inequities. First, we found that plurisexual youth had the highest inequities in substance use compared to monosexual youth (e.g. heterosexual, lesbian and gay). Second, we found that inequities in substance use were greater among SMY girls and young women compared to SMY boys and young men. These finding are consistent with the prior meta‐analysis of substance use disparities [13] and other studies demonstrating that plurisexual and SMY girls and young women have elevated rates of numerous poor health outcomes [5, 41]. Third, our findings demonstrate developmental differences, where inequities were more pronounced in adolescence compared to young adulthood. Given that prior longitudinal research has shown that substance use inequities have increased, particularly among sexual minority girls and young women [15, 42], and more recent work has indicated potentially elevated rates for SMY of color [18, 19, 22], prevention and intervention research is urgently needed to address the unique biopsychosocial and health needs of these particular subgroups.

Several etiological factors shape SMY's substance use. According to the oppression framework, inequities in substance use are rooted in systems of oppression [6]. SMY experience and navigate pervasive oppression throughout their development (e.g. heterosexist‐ and anti‐bisexual‐based victimization or discrimination, identity concealment, internalized oppression and heterosexist laws) [5, 6, 43, 44]. Therefore, SMY may use substances to cope with distress, shame, harm and lack of safety inflicted by systems of oppression and/or to resist oppression (e.g. use substances to be in community, celebrate, feel liberated and be sexual) [6, 20, 45, 46]. Furthermore, oppression shapes cultural and social norms related to substance use, which then create permissive substance use norms in the lesbian, gay, bisexual, trans, queer (LGBTQ) community [6, 45, 47, 48]. In fact, studies have documented that alcohol and nicotine companies specifically target the LGBTQ community [6, 49], creating further risk for hazardous substance use among SMY. Last, common etiological factors that influence substance use and development of SUDs (e.g. traumatic life experiences, genetic factors) among the broader youth population also play a role for SMY and intersect with their experiences with oppression [6, 20].

Some methodological factors played a role in our findings. Inequities in substance use were elevated when lifetime versus recent substance use was assessed. This finding is unsurprising as substance use experimentation is more common in adolescence than recent or repeated use. Therefore, more SMY and heterosexual youth would have had an opportunity to use, further magnifying the disparity. Additionally, this finding might suggest that SMY are more likely to experiment in substance use, which theory suggests is because of oppression‐based stress experiences and differences in substance use norms and willingness to use substances [5, 6, 45, 47, 48]. Although underpowered to estimate the magnitude of inequities along the attraction dimension of sexual orientation, our results also indicated that substance use inequities were documented across both identity and behavioral dimensions of sexual orientation. Adolescence is a hallmark period of identity exploration and development [41], therefore, assessing only sexual identification or behavior may miss youth who have not yet identified or do not feel comfortable identifying as a sexual minority, but may be aware of their attractions. These results highlight the importance of comprehensive assessment of sexual orientation among youth, including fluidity [50], especially as only a few studies measured more than one sexual orientation dimension.

The findings of this study have research and clinical implications. Additional research is needed to examine developmental and etiological factors that impact substance use initiation. More longitudinal studies are needed to document inequities and their trajectories from adolescence and throughout the lifespan. Moreover, effective intervention and prevention strategies targeting substance use among SMY are lacking [5,41]. SMY face several barriers when they are in treatment, including providers' inadequate cultural competency, lack of autonomy over their own treatment and fears of confidentiality (e.g. providers outing SMY to their parents/guardians) [51]. Therefore, policies and interventions that increase access to SUD treatment and increase cultural humility among providers are needed. Furthermore, routine screenings for harmful substance use across settings (e.g. schools, primary care, emergency departments and community organizations) and other universal prevention interventions may help prevent and identify harmful substance use among SMY. However, screenings must also be coupled with accessible, de‐stigmatizing, culturally affirming and confidential services to ensure their success and in order not pathologize or harm youth who engage in hazardous substances use. Last, multi‐level interventions that increase affirmation, resilience, resistance and intervene on oppression are essential to reducing and eliminating inequities in substance use [6, 44]. These interventions should target oppression and stigma targeting SMY (e.g. heterosexism, anti‐bisexual prejudice) as well as stigma related to substance use and addiction that marginalizes youth who use substances or have SUDs.

This study has some noteworthy limitations. Although SMY consistently showed elevated substance use compared to their heterosexual peers, the substantial heterogeneity observed in our primary analyses (I^2^ > 90% across most substance outcomes in Table 2) indicates considerable variation in effect sizes across studies. Similarly, the wide prediction intervals suggest that the magnitude of disparities varies depending on different populations, settings and study characteristics. Although we accounted for sampling differences through moderator analyses, the integration of effect sizes derived from weighted estimates of probability samples versus estimates from convenience samples was a methodological limitation in our meta‐analysis. Our moderator analyses were limited to bivariate models examining one variable at a time, which may yield confounded associations when moderators are correlated, particularly when interpreting differences because of study‐level characteristics. Moreover, because of resource constraints, our search was limited to studies in published in English, limiting our understanding of published work in other languages. Although we assessed the percentage of transgender participants in studies where gender identity was reported, these studies were quite limited, reducing our power to adequately test for gender identity effects. Similarly, studies were not clear on whether they assessed sex or gender, as such, our findings mirror limitations found in the reporting of primary studies that conflate sex and gender. Last, although we examined proxies for study quality, such as study sampling strategy (probability vs. convenience sampling), publication type (published studies vs. dissertation/thesis) and measurement duration (lifetime vs. recent use), a formal assessment of study quality using a standardized tool was not conducted because of limited resources, especially given the large number of studies included.

This systematic review and meta‐analysis provide the most rigorous and comprehensive evidence documenting significant and consistent sexual orientation inequities in substance use among youth, indicating that SMY have significantly elevated rates of substance use compared to their heterosexual peers. Multi‐level approaches are needed to reduce and eliminate these inequities, especially targeting oppression, increasing safety and affirmation and improving access to culturally affirming treatment for SMY.

## AUTHOR CONTRIBUTIONS

**Ethan H. Mereish:** Conceptualization; data curation; funding acquisition; investigation; methodology; project administration; supervision; writing—original draft; writing—review and editing. **Hyemin Lee:** Conceptualization; data curation; formal analysis; methodology; writing—original draft; writing—review and editing. **Jeremy T. Goldbach:** Conceptualization; methodology; writing—review and editing. **Sophie Hathaway:** Data curation; writing—review and editing. **Emily A. Hennessy:** Formal analysis; investigation; methodology; supervision; writing—review and editing.

## DECLARATION OF INTERESTS

None.

## Supporting information


**Table S1.** Meta‐regression of effect sizes on standard errors using Robust Variance Estimation
**Figure S1.** Number of included studies by publication year
**Figure S2.** Contour‐enhanced funnel plot for continuous outcomes
**Figure S3.** Contour‐enhanced funnel plot for dichotomous outcomes.

## Data Availability

The data that support the findings of this study are openly available through the Open Science Framework at: https://osf.io/t6ywu/files/osfstorage.

## Footnotes

^1^
For mixed/polysubstance use, when studies reported multiple effect sizes, one was selected per study based on: (1) largest sample size to maximize precision; and (2) age most similar to other studies for one longitudinal study with equal sample sizes at multiple time points. For age of initiation for continuous outcomes, only cannabis initiation could be synthesized (k = 3). Additional individual effect sizes for other substance initiation were calculated, but not pooled because of conceptual heterogeneity: Kecojevic *et al.* (2012): opioid (g = −0.18, SE = 0.04), tranquilizer (g = −0.28, SE = 0.04), stimulant (g = −0.18, SE = 0.04), cocaine (g = −0.08, SE = 0.04), methamphetamine (g = 0.03, SE = 0.04), heroin (g = 0.04, SE = 0.04) and crack (g = 0.00, SE = 0.04) initiation; Talley et al. (2019): alcohol (g = −1.92, SE = 0.14) and tobacco (g = −1.92, SE = 0.14) initiation.

^2^
We also assessed the percentage of transgender participants reported in each sample; however, due to small sample sizes, we were not powered to test this as a moderator.

3 Given that many studies were not clear on whether they assessed sex or gender, we use “sex/gender” language here in the results to be inclusive of references to sex assigned at birth or gender.
