# Supplementary material for: A meta‐analysis of sexual orientation inequities in substance use among youth
Source: Addiction. 2025 Dec 24;121(5):1048–63. doi: 10.1111/add.70301 (PMC13088934; doi:10.1111/add.70301)
Supplement: Supplementary file 1 — Table S1. Meta‐regression of effect sizes on standard errors using Robust Variance Estimation Figure S1. Number of included studies by publication year Figure S2. Contour‐enhanced funnel plot for continuous outcomes Figure S3. Contour‐enhanced funnel plot for dichotomous outcomes. [file ADD-121-1048-s001.pdf]

List of Supplemental Files

1. Appendix S1. Literature Search Strategies

2. Appendix S2. Reference list of included studies

3. Appendix S3. Characteristics of included studies – Please see "Study_Summary" sheet in "OSF_Datafile_Study_Effect_Sizes_09.08.25" spreadsheet in the "Data Files and Data Analysis Code" folder, which can be accessed here: https://osf.io/t6ywu/files/osfstorage

4. Table S1. Meta-regression of effect sizes on standard errors using Robust Variance Estimation

5. Figure S1. Number of included studies by publication year

6. Figure S2. Contour-enhanced funnel plot for continuous outcomes

7. Figure S3. Contour-enhanced funnel plot for dichotomous outcomes

Supplemental Online Content

Appendix S1. Literature Search Strategies

1. **Pubmed**

- Sexual minority

Title/Abstract:
Gay OR "Men who have sex with men" OR "Men who have sex with men and women" OR Homosexual* OR Lesbian OR "Women who have sex with women" OR "Women who have sex with women and men" OR Bisexual* OR Pansexual OR Asexual OR "LGBT people" OR "Sexual minority" OR LGBT* OR Queer OR "Homosexual male" OR "Homosexual female" OR "Homosexual men" OR "Homosexual women" OR "Mostly heterosexual women" OR "Mostly heterosexual men" OR "Same-sex attraction" OR "Sexual orientation" OR "Sexual identity" OR Lesbianism OR "Women loving women" OR Heteroflexible OR MSM OR WSW OR MSMW OR WSWM OR LGB OR GLB OR GLBT OR Same-sex OR "Sexual attraction" OR "Men loving men" OR Heterosexual OR "Opposite-sex attraction" OR Sexuality OR "Sexual minorit*"

MeSH Terms:
Sexual Minorities OR Bisexuality OR Homosexuality, Female OR Homosexuality, Male OR Homophobia

- Substance use

Title/Abstract:
Alcohol* OR "Binge drink" OR Drink* OR Ethanol OR "Risky drinking" OR Drug* OR Marijuana OR Cannabi* OR Sedative* OR Benzodiazepine* OR Cocaine OR Stimulant* OR Amphetamine* OR Methamphetamine OR "Bath salt*" OR PCP OR Phencyclidine OR Ecstasy OR MDMA OR 3,4-Methylenedioxymethamphetamine OR GHB OR "Gamma hydroxybutyrate" OR Ketamine OR LSD OR "Lysergic acid diethylamide" OR Opiate* OR Opioid* OR Heroin OR Fentanyl OR Morphine OR Oxycodone OR Hydrocodone OR "Prescription drug*" OR "Prescription drug abuse" OR Inhalant* OR Addict* OR Dependen* OR "Drug abuse" OR "Substance use" OR "Substance abuse" OR "Substance misuse" OR K2 OR Narcotic* OR Psychodelic OR "Glue sniffing" OR "Analgesic agent abuse" OR Doping OR "Drug misuse" OR Phenethylamine OR Lysergide OR "E-cigarette*" OR "Electronic cigarette*" OR "Electronic nicotine delivery" OR Vape* OR Vapi* OR Tobacco OR Cigarette* OR Nicotine OR Smok* OR "Chewing tobacco" OR Hookah OR Shisha OR Snuff

MeSH Terms:
Ethanol OR Alcohols OR Alcohol-Induced Disorders OR Alcohol-Related Disorders OR Alcohol Drinking OR Alcohol Drinking in College OR Underage Drinking OR Alcoholism OR Substance-Related Disorders OR Substance Abuse, Intravenous OR Prescription Drug Misuse OR Addictive Behavior OR Opioid-Related Disorders OR Cocaine-Related Disorders OR Cocaine OR Crack Cocaine OR Amphetamine-Related Disorders OR Amphetamine OR Amphetamines OR Methamphetamine OR Morphine Dependence OR Heroin Dependence OR Heroin OR Analgesics, Opioid OR Opiate Substitution Treatment OR Cannabis OR Marijuana Abuse OR Marijuana Smoking OR Benzodiazepines OR Hypnotics and Sedatives OR Phencyclidine Abuse OR N-Methyl-3,4 OR Methylenedioxyamphetamine OR Hallucinogens OR Ketamine OR Lysergic Acid Diethylamide OR Fentanyl OR Inhalant abuse

- Youth

Title/Abstract:
Teen* OR Youth* OR Adolescen* OR “Young adult*” OR “Emerging adult*” OR “Adolescent" OR “Young people” OR “Adolescent development” OR “College students” OR “High school students” OR “Middle school students” OR Students OR Juvenile

MeSH Terms:
Adolescent OR Young Adult

2. **APA PsycINFO**

- Sexual minority

Gay OR “Men who have sex with men” OR “Men who have sex with men and women” OR Homosexual* OR Lesbian OR “Women who have sex with women” OR “Women who have sex with women and men” OR Bisexual* OR Pansexual OR Asexual OR “LGBT people” OR “Sexual minority” OR LGBT* OR Queer OR “Homosexual male” OR “Homosexual female” OR “Homosexual men” OR “Homosexual women” OR “Mostly heterosexual women” OR “Mostly heterosexual men” OR “Same-sex attraction” OR “Sexual orientation” OR “Sexual identity” OR Lesbianism OR “Women loving women” OR Heteroflexible OR MSM OR WSW OR MSMW OR WSWM OR LGB OR GLB OR GLBT OR Same-sex OR “Sexual attraction” OR “Men loving men” OR Heterosexual OR “Opposite-sex attraction” OR Sexuality OR “Sexual minorit*”

- Substance use

Alcohol* OR “Binge drink*” OR Drink* OR Ethanol OR “Risky drink*” OR Drug* OR Marijuana OR Cannabi* OR Sedative* OR Benzodiazepine* OR Cocaine OR Stimulant* OR Amphetamine* OR Methamphetamine OR “Bath salt*” OR PCP OR Phencyclidine OR Ecstasy OR MDMA OR Methylenedioxymethamphetamine OR GHB OR “Gamma hydroxybutyrate” OR Ketamine OR LSD OR “Lysergic acid diethylamide” OR Opiate* OR Opioid* OR Heroin OR Fentanyl OR Morphine OR Oxycodone OR Hydrocodone OR “Prescription drug*” OR “Prescription drug abuse” OR Inhalant* OR Addict* OR Dependen* OR “Drug abuse OR Substance* OR “Substance abuse OR “Substance misuse” OR K2 OR Narcotic* Or Psychodelic OR “Glue sniffing” OR “Analgesic agent abuse” OR Doping OR “Drug misuse” OR Phenethylamine OR Lysergide OR E-cigarette* OR “Electronic cigarette*” OR “Electronic nicotine delivery” OR Vape* OR Vapi* OR Tobacco OR Cigarette* OR Nicotine OR Smok* OR “Chewing tobacco” OR Hookah OR Shisha OR Snuff

- Youth

Teen* OR Youth* OR Adolescen* OR “Young adult” OR “Emerging adult” OR Adolescent OR “Young people” OR “Adolescent development” OR “College students” OR “High school students” OR “Middle school students” OR Students OR Juvenile

3. **Web of Science**

- Sexual minority

TS=Gay OR TS=“Men who have sex with men” OR TS=“Men who have sex with men and women” OR TS=Homosexual* OR TS=Lesbian OR TS=“Women who have sex with women” OR TS= “Women who

have sex with women and men" OR TS=Bisexual* OR TS=Pansexual OR TS=Asexual OR TS="LGBT people" OR TS="Sexual minority" OR TS=LGBT* OR TS=Queer OR TS=(Homosexual AND Male) OR TS=(Homosexual AND Female) OR TS=(Homosexual AND Men) OR TS=(Homosexual AND Women) OR TS=(Mostly AND Heterosexual AND Women) OR TS=(Mostly AND Heterosexual AND Men) OR TS=("Same sex" AND Attraction) OR TS=(Sexual AND Orientation) OR TS=(Sexual AND Identity) OR TS=Lesbianism OR TS=(Women AND Loving AND Women) OR TS=Heteroflexible OR TS=MSM OR TS=WSW OR TS=MSMW OR TS=WSWM OR TS=LGB OR TS=GLB OR TS=GLBT OR TS="Same sex" OR TS="Sexual attraction" OR TS=(Men AND Loving AND Men) OR TS=Heterosexual OR TS=("Opposite-sex" AND Attraction) OR TS=Sexuality OR TS=(Sexual AND Minorit*)

- Substance use

TS=Alcohol* OR TS="Binge drink*" OR TS=Drink* OR TS=Ethanol OR TS="Risky drinking" OR TS=Drug* OR TS=Marijuana OR TS=Cannabi* OR TS=Sedative* OR TS=Benzodiazepine* OR TS=Cocaine OR TS=Stimulant* OR TS=Amphetamine* OR TS=Methamphetamine OR TS="Bath salt*" OR TS=PCP OR TS=Phencyclidine OR TS=Ecstasy OR TS=MDMA OR TS=3,4-Methylenedioxymethamphetamine OR TS=GHB OR TS="Gamma hydroxybutyrate" OR TS=Ketamine OR TS=LSD OR TS="Lysergic acid diethylamide" OR TS=Opiate* OR TS=Opioid* OR TS=Heroin OR TS=Fentanyl OR TS=Morphine OR TS=Oxycodone OR TS=Hydrocodone OR TS= "Prescription drug*" OR TS="Prescription drug abuse" OR TS=Inhalant* OR TS=Addict* OR TS=Dependen* OR TS="Drug abuse" OR TS="Substance use" OR TS="Substance abuse" OR TS="Substance misuse" OR TS=K2 OR TS=Narcotic* OR TS=Psychodelic OR TS="Glue sniffing" OR TS="Analgesic agent abuse" OR TS=Doping OR TS="Drug misuse" OR TS=Phenethylamine OR TS=Lysergide OR TS=E-cigarette* OR TS="Electronic cigarette*" OR TS="Electronic nicotine delivery" OR TS=Vape* OR TS=Vapi* OR TS=Tobacco OR TS=Cigarette* OR TS=Nicotine OR TS=Smok* OR TS="Chewing tobacco" OR TS=Hookah OR TS=Shisha OR TS=Snuff

- Youth

TS=Teen* OR TS=Youth* OR TS=Adolescen* OR TS="Young adult" OR TS="Emerging adult" OR TS="Adolescent" OR TS="Adolescence" OR TS="Young people" OR TS="Adolescent development" OR TS="College students" OR TS="High school students" OR TS="Middle school students" OR TS=Students OR TS=Juvenile

4. **ProQuest**

- Sexual minority

Gay OR "Men who have sex with men" OR "Men who have sex with men and women" OR Homosexual* OR Lesbian OR "Women who have sex with women" OR "Women who have sex with women and men" OR Bisexual* OR Pansexual OR Asexual OR "LGBT people" OR "Sexual minority" OR LGBT* OR Queer OR "Homosexual male" OR "Homosexual female" OR "Homosexual men" OR "Homosexual women" OR "Mostly heterosexual women" OR "Mostly heterosexual men" OR "Same-sex attraction" OR "Sexual orientation" OR "Sexual identity" OR Lesbianism OR "Women loving women" OR Heteroflexible OR MSM OR WSW OR MSMW OR WSWM OR LGB OR GLB OR GLBT OR Same-sex OR "Sexual attraction" OR "Men loving men" OR Heterosexual OR "Opposite-sex attraction" OR Sexuality OR "Sexual minorit*"

- Substance use

Alcohol* OR “Binge drink*” OR Drink* OR Ethanol OR “Risky drink*” OR Drug* OR Marijuana OR Cannabi* OR Sedative* OR Benzodiazepine* OR Cocaine OR Stimulant* OR Amphetamine* OR Methamphetamine OR “Bath salt*” OR PCP OR Phencyclidine OR Ecstasy OR MDMA OR Methylenedioxymethamphetamine OR GHB OR “Gamma hydroxybutyrate” OR Ketamine OR LSD OR “Lysergic acid diethylamide” OR Opiate* OR Opioid* OR Heroin OR Fentanyl OR Morphine OR Oxycodone OR Hydrocodone OR “Prescription drug*” OR “Prescription drug abuse” OR Inhalant* OR Addict* OR Dependen* OR “Drug abuse” OR Substance* OR “Substance abuse” OR “Substance misuse” OR K2 OR Narcotic* Or Psychodelic OR “Glue sniffing” OR “Analgesic agent abuse” OR Doping OR “Drug misuse” OR Phenethylamine OR Lysergide OR E-cigarette* OR “Electronic cigarette*” OR “Electronic nicotine delivery” OR Vape* OR Vapi* OR Tobacco OR Cigarette* OR Nicotine OR Smok* OR “Chewing tobacco” OR Hookah OR Shisha OR Snuff

- Youth

Teen* OR Youth* OR Adolescen* OR “Young adult” OR “Emerging adult” OR Adolescent OR “Young people” OR “Adolescent development” OR “College students” OR “High school students” OR “Middle school students” OR Students OR Juvenile

Supplemental Online Content

Appendix S2. Reference list of included studies (n = 304)

1. Agnich LE, Stogner JM, Miller BL, Marcum CD. Purple drank: Prevalence and characteristics of misusers of codeine cough syrup mixtures. *Addict Behav*. 2013;38(9):2445-2449.
2. Allen ST, Ruiz MS, O'Rourke A. Differences in the prevalence of risk behaviors between heterosexual and lesbian, gay, bisexual, and questioning (LGBQ) female adolescents in the juvenile justice system. *J Gay Lesbian Soc Serv*. 2016;28(2):171-175.
3. Ancheta AJ, Caceres BA, Jackman KB, Kreuze E, Hughes TL. Sexual identity differences in health behaviors and weight status among urban high school students. *Behav Med*. 2021;47(4):259-271.
4. Assis SG, Gomes R, Pires TO. Adolescence, sexual behavior and risk factors to health. *Rev Saude Publica*. 2014;48(1):43-51.
5. Austin EL, Bozick R. Sexual orientation, partnership formation, and substance use in the transition to adulthood. *J Youth Adolesc*. 2012;41(2):167-178.
6. Austin SB, Rosario M, McLaughlin KA, et al. Sexual orientation and diurnal cortisol patterns in a cohort of U.S. young adults. *Psychoneuroendocrinology*. 2016;69:197-208.
7. Azagba S, Shan L. Disparities in the frequency of tobacco products use by sexual identity status. *Addict Behav*. 2021;122:107032.
8. Azagba S, Shan L. Tobacco craving, nicotine dependence, and quit intentions among LGB and non-LGB high school students: a quasi-experimental analysis. *Int J Environ Res Public Health*. 2021;18(17):9000.
9. Azagba S, Ebling T, Korkmaz A. Disposable e-cigarette use: Factors, frequency and cigarette smoking among United States high school students. *Addiction*. 2024.
10. Azagba S, Ebling T, Shan L. Sexual minority youth e-cigarette use. Pediatrics. 2023;151(3):e2022058414.
11. Azagba S, Asbridge M, Langille D, Baskerville B. Disparities in tobacco use by sexual orientation among high school students. *Prev Med*. 2014;69:307-311.
12. Azagba S, Latham K, Shan L. Cigarette smoking, e-cigarette use, and sexual identity among high school students in the USA. *Eur J Pediatr*. 2019;178(9):1343-1351.
13. Ballard ME, Jameson JP, Martz DM. Sexual identity and risk behaviors among adolescents in rural Appalachia. *J Rural Ment Health*. 2017;41(1):17-29.
14. Baranauskas M, Kupčiūnaitė I, Stukas R. Psychoactive substance effect on mental health and well-being focusing on student-aged Lithuanian cohort of sexual minorities. *Sustainability*. 2022;14(20):13063.
15. Beard JC. LGB youth's risky sexual behavior: protective factors and gender differences [dissertation]. Urbana, IL: University of Illinois Urbana-Champaign; 2013.
16. Becker M, Cortina KS, Tsai YM, Eccles JS. Sexual orientation, psychological well-being, and mental health: A longitudinal analysis from adolescence to young adulthood. *Psychol Sex Orient Gend Divers*. 2014;1(2):132-145.
17. Benz MB, Reed KP, Bishop LS. Stigma and help-seeking: The interplay of substance use and gender and sexual minority identity. *Addict Behav*. 2019;97:63-69.
18. Berg CJ, Duan X, Romm K, et al. Young adults' vaping, readiness to quit, and recent quit attempts: The role of co-use with cigarettes and marijuana. *Nicotine Tob Res*. 2021;23(6):1019-1029.
19. Berg CJ, Romm KF, Patterson B, Wysota CN. Heated tobacco product awareness, use, and perceptions in a sample of young adults in the United States. *Nicotine Tob Res*. 2021;23(11):1967-1971.
20. Bernstein MH, Stein LAR. Do bisexual girls report higher rates of substance use than heterosexual girls? A failure to replicate with incarcerated and detained youth. *J Bisex*. 2015;15(4):498-508.

21. Bhatia D, Berg O, Davies R, Mikulich Gilbertson S, Sakai J. The association between sexual identity, depression, and adolescent substance use. *Child Psychiatry Hum Dev*. 2023:1-8.
22. Birkett M, Espelage DL, Koenig B. LGB and questioning students in schools: the moderating effects of homophobic bullying and school climate on negative outcomes. *J Youth Adolesc*. 2009;38(7):989-1000.
23. Bishop MD, Ioverno S, Russell ST. Sexual minority youth's mental health and substance use: The roles of victimization, cybervictimization, and non-parental adult support. *Curr Psychol*. 2023;42(6):5075-5087.
24. Blashill AJ, Safren SA. Sexual orientation and anabolic-androgenic steroids in U.S. adolescent boys. *Pediatrics*. 2014;133(3):469-475.
25. Blashill AJ, Calzo JP, Griffiths S, Murray SB. Anabolic steroid misuse among US adolescent boys: Disparities by sexual orientation and race/ethnicity. *Am J Public Health*. 2017;107(2):319-321.
26. Blosnich JR, Horn K. Associations of discrimination and violence with smoking among emerging adults: differences by gender and sexual orientation. *Nicotine Tob Res*. 2011;13(12):1284-1295.
27. Blosnich J, Jarrett T, Horn K. Racial and ethnic differences in current use of cigarettes, cigars, and hookahs among lesbian, gay, and bisexual young adults. *Nicotine Tob Res*. 2011;13(6):487-491.
28. Blosnich J, Jarrett T, Horn K. Disparities in smoking and acute respiratory illnesses among sexual minority young adults. *Lung*. 2010;188(5):401-407.
29. Bluestein BM. The associations between sexual orientation and psychopathology, substance use, and experiences of violence: Results from a nationally representative longitudinal study [dissertation]. East Lansing, MI: Michigan State University; 2010.
30. Bonny-Noach H, Shechory-Bitton M. Differences in substance use by sexual orientation and gender among Jewish young adults in Israel. *Isr J Health Policy Res*. 2020;9:1-10.
31. Bos HM, van Beusekom G, Sandfort TG. Drinking motives, alcohol use, and sexual attraction in youth. *J Sex Res*. 2016;53(3):309-312.
32. Bowring AL, Vella AM, Degenhardt L, Hellard M, Lim MS. Sexual identity, same-sex partners and risk behaviour among a community-based sample of young people in Australia. *Int J Drug Policy*. 2015;26(2):153-161.
33. Brewster KL, Tillman KH. Sexual orientation and substance use among adolescents and young adults. *Am J Public Health*. 2012;102(6):1168-1176.
34. Brittain DR, Dinger MK. An examination of health inequities among college students by sexual orientation identity and sex. *J Public Health Res*. 2015;4(1):1-6.
35. Bruce D, Kahana SY, Bauermeister JA, et al. Neighborhood-level and individual-level correlates of cannabis use among young persons living with HIV/AIDS. *Drug Alcohol Depend*. 2015;151:173-180.
36. Busseri MA, Willoughby T, Chalmers H, Bogaert AF. On the association between sexual attraction and adolescent risk behavior involvement: Examining mediation and moderation. *Dev Psychol*. 2008;44(1):69-80.
37. Buttazzoni A, Tariq U, Thompson-Haile A, Burkhalter R, Cooke M, Minaker L. Adolescent gender identity, sexual orientation, and cannabis use: potential mediations by internalizing disorder risk. *Health Educ Behav*. 2021;48(1):82-92.
38. Button DM, O'Connell DJ, Gealt R. Sexual minority youth victimization and social support: the intersection of sexuality, gender, race, and victimization. *J Homosex*. 2012;59(1):18-43.
39. Calzo JP, Turner BC, Marro R, Phillips GL II. Alcohol Use and Disordered Eating in a US Sample of Heterosexual and Sexual Minority Adolescents. *J Am Acad Child Adolesc Psychiatry*. 2019;58(2):200-210.
40. Caputi TL. Sex and orientation identity matter in the substance use behaviors of sexual minority adolescents in the United States. *Drug Alcohol Depend*. 2018;187:142-148.

41. Caputi TL, Smith LR, Strathdee SA, Ayers JW. Substance Use Among Lesbian, Gay, Bisexual, and Questioning Adolescents in the United States, 2015. *Am J Public Health*. 2018;108(8):1031-1034.
42. Chakraborty P, Alalwan M, Johnson RM, Li L, Lancaster KE, Zhu M. Mental health and substance use by sexual minority status in high school students who experienced sexual violence. *Ann Epidemiol*. 2021;64:127-131.
43. Chassman S, Barman-Adhikari A, Hsu HT, et al. Prevalence and correlates of illicit substance use among young adults experiencing homelessness in seven cities across the United States. *J Drug Issues*. 2022;52(4):488-508.
44. Chen DT, Girvalaki C, Filippidis FT. Disposable e-cigarette use and associated factors in US middle and high school students, 2021–2022. *Tob Induc Dis*. 2024;22:117.
45. Chen YT, Issema RS, Khanna AS, Pho MT, Schneider JA, UConnect Study Team. Prescription Opioid Use in a Population-Based Sample of Young Black Men Who Have Sex with Men: A Longitudinal Cohort Study. *Subst Use Misuse*. 2019;54(12):1991-2000.
46. Cheng CHE, Gipson JD, Perez TL, Cochran SD. Same-Sex Behavior and Health Indicators of Sexually Experienced Filipino Young Adults. *Arch Sex Behav*. 2016;45(6):1471-1482.
47. Chien YS, Schwartz G, Huang L, Kawachi I. State LGBTQ policies and binge drinking among sexual minority youth in the US: a multilevel analysis. *Soc Psychiatry Psychiatr Epidemiol*. 2022;57(1):183-194.
48. Choi EPH, Wong JYH, Lo HHM, Wong W, Chio JHM, Fong DYT. Association Between Using Smartphone Dating Applications and Alcohol and Recreational Drug Use in Conjunction With Sexual Activities in College Students. *Subst Use Misuse*. 2017;52(4):422-428.
49. Clary KL, Goffnett J, Bennett K, Smith D. A comparison of developmental reasons for substance use between sexual minority and heterosexual emerging adults. *J Gay Lesbian Soc Serv*. 2022;34(3):360-380.
50. Clayton HB, Andrzejewski J, Johns MM, Lowry R, Ashley C. Does the association between substance use and sexual risk behaviors among high school students vary by sexual identity? *Addict Behav*. 2019;93:122-128.
51. Coble CA, Silver EJ, Chhabra R. Description of sexual orientation and sexual behaviors among high school girls in New York City. *J Pediatr Adolesc Gynecol*. 2017;30(4):460-465.
52. Comulada WS, Rotheram-Borus MJ, Arnold EM, et al. Using machine learning to identify predictors of sexually transmitted infections over time among young people living with or at risk for HIV who participated in ATN protocols 147, 148, and 149. *Sex Transm Dis*. 2023;50(11):739-745.
53. Corliss HL, Rosario M, Wypij D, Fisher LB, Austin SB. Sexual orientation and drug use in a longitudinal cohort study of US adolescents. *Addict Behav*. 2010;35(5):517-521.
54. Corliss HL, Wadler BM, Jun HJ, et al. Sexual-orientation disparities in cigarette smoking in a longitudinal cohort study of adolescents. *Nicotine Tob Res*. 2013;15(1):213-222.
55. Corliss HL, Rosario M, Birkett MA, Newcomb ME, Buchting FO, Matthews AK. Sexual orientation disparities in adolescent cigarette smoking: intersections with race/ethnicity, gender, and age. *Am J Public Health*. 2014;104(6):1137-1147.
56. Cotter P, Corcoran P, McCarthy J, et al. Victimisation and psychosocial difficulties associated with sexual orientation concerns: a school-based study of adolescents. *Ir Med J*. 2014;107(10):310-313.
57. Coulter RWS, Birkett M, Corliss HL, Hatzenbuehler ML, Mustanski B, Stall RD. Associations between LGBTQ-affirmative school climate and adolescent drinking behaviors. *Drug Alcohol Depend*. 2016;161:340-347.
58. Coulter RWS, Marzell M, Saltz R, Stall R, Mair C. Sexual-orientation differences in drinking patterns and use of drinking contexts among college students. *Drug Alcohol Depend*. 2016;160:197-204.

59. Coulter RWS, Jun HJ, Calzo JP, et al. Sexual-orientation differences in alcohol use trajectories and disorders in emerging adulthood: results from a longitudinal cohort study in the United States. *Addiction*. 2018;113(9):1619-1632.
60. Coulter RWS, Bersamin M, Russell ST, Mair C. The effects of gender- and sexuality-based harassment on lesbian, gay, bisexual, and transgender substance use disparities. *J Adolesc Health*. 2018;62(6):688-700.
61. Coulter RWS, Ware D, Fish JN, Plankey MW. Latent classes of polysubstance use among adolescents in the United States: intersections of sexual identity with sex, age, and race/ethnicity. *LGBT Health*. 2019;6(3):116-125.
62. Crawford TN, Ridner SL. Differences in well-being between sexual minority and heterosexual college students. *J LGBT Youth*. 2018;15(3):243-255.
63. Curry CW, Beach LB, Wang X, et al. At-school victimization and alcohol use among minoritized US youth, 2009–2017. *Am J Prev Med*. 2022;63(5):772-782.
64. Cusack CE, Christian C, Drake JE, Levinson CA. A network analysis of eating disorder symptoms and co-occurring alcohol misuse among heterosexual and sexual minority college women. *Addict Behav*. 2021;118:106867.
65. Cutuli JJ, Treglia D, Herbers JE. Adolescent homelessness and associated features: prevalence and risk across eight states. *Child Psychiatry Hum Dev*. 2020;51(1):48-58.
66. D'Agati D, Kahn G, Swartz KL. Preteen behaviors and sexual orientation of high school students who experience sexual violence, United States, 2015-2017. *Public Health Rep*. 2021;136(2):132-135.
67. da Graça Alves AF. Sexual violence and substance use among Portuguese college students: mental health implications [master's thesis]. Universidade da Beira Interior; 2023.
68. Dagirmanjian FR, McDaniel AE, Shadick R. Sexual orientation and college students' reasons for nonmedical use of prescription drugs. *Subst Use Misuse*. 2017;52(8):1011-1018.
69. Dai H. Tobacco product use among lesbian, gay, and bisexual adolescents. *Pediatrics*. 2017;139(4):e20163276.
70. Dai HD, Leventhal AM. Use of traditional smokeless, snus, and dissolvable tobacco among US youth. *Am J Prev Med*. 2024;64(2):204-212.
71. Dai HD, Subramanian R, Mahroke A, Wang M. Prevalence and factors associated with vaping cannabidiol among US adolescents. *JAMA Netw Open*. 2023;6(8):e2329167.
72. Dai HY, Ingram DG, Taylor JB. Hierarchical and mediation analysis of disparities in very short sleep among sexual minority youth in the US, 2015. *Behav Sleep Med*. 2020;18(4):433-446.
73. De Genna NM, Coulter RWS, Goldschmidt L, Boss N, Hossain F, Richardson GA. Prenatal substance use among young pregnant sexual minority people. *LGBT Health*. 2024;11(1):74-79.
74. De Pedro KT, Shim-Pelayo H. Prevalence of substance use among lesbian, gay, bisexual, and transgender youth in military families: findings from the California Healthy Kids Survey. *Subst Use Misuse*. 2018;53(8):1372-1376.
75. Demant D, Hides L, Kavanagh DJ, White KM, Winstock AR, Ferris J. Differences in substance use between sexual orientations in a multi-country sample: findings from the Global Drug Survey 2015. *J Public Health (Oxf)*. 2017;39(3):532-541.
76. Depa N, Desai S, Patel S, et al. Mental health disparities amongst sexual-minority adolescents of the US–A national survey study of YRBSS-CDC. *Psychiatry Res*. 2022;314:114635.
77. Dermody SS. Risk of polysubstance use among sexual minority and heterosexual youth. *Drug Alcohol Depend*. 2018;192:38-44.

78. Dermody SS, Marshal MP, Cheong J, Chung T, Stepp SD, Hipwell A. Adolescent sexual minority girls are at elevated risk for use of multiple substances. *Subst Use Misuse*. 2016;51(5):574-585.
79. Dermody SS, McGinley J, Eckstrand K, Marshal MP. Sexual minority female youth and substance use disparities across development. *J LGBT Youth*. 2019;17(2):214-229.
80. Donahue K, Långström N, Lundström S, Lichtenstein P, Forsman M. Familial factors, victimization, and psychological health among sexual minority adolescents in Sweden. *Am J Public Health*. 2017;107(2):322-328.
81. Donaldson CD, Fecho CL, Ta T, et al. Vaping identity in adolescent e-cigarette users: a comparison of norms, attitudes, and behaviors. *Drug Alcohol Depend*. 2021;223:108712.
82. Donaldson CD, Stupplebeen DA, Couch ET, et al. Perceived discrimination and youth vaping: The role of intersectional identities. *Drug Alcohol Depend*. 2024;260:111313.
83. Donaldson CD, Stupplebeen DA, Wilkinson ML, Zhang X, Williams RJ. Intersectional disparities in youth tobacco use by sexual and/or gender minority status and race and/or ethnicity. *Nicotine Tob Res*. 2023;25(5):898-907.
84. Doxbeck CR. Old school, new stress: examining the intersection of sexual identity, student experiences, and negative outcomes [dissertation]. Buffalo, NY: State University of New York at Buffalo; 2022.
85. Doxbeck CR, Jaeger JA, Bleasdale JM. Understanding pathways to e-cigarette use across sexual identity: A multi-group structural equation model. *Addict Behav*. 2021;114:106748.
86. Duangchan C, Matthews AK, Smith AU, Steffen AD. Sexual minority status, school-based violence, and current tobacco use among youth. *Tob Prev Cessat*. 2022;8:24.
87. Dunbar MS, Siconolfi D, Rodriguez A, et al. Alcohol use and cannabis use trajectories and sexual/gender minority disparities in young adulthood. *Psychol Addict Behav*. 2022;36(5):477.
88. Duryea DG, Frantz TT. An examination of drinkers' consequences by sexual orientation. *J Am Coll Health*. 2011;59(7):649-654.
89. Duryea DG, Calleja NG, MacDonald DA. Nonmedical use of prescription drugs by college students with minority sexual orientations. *J College Student Psychother*. 2015;29(2):147-159.
90. Edwards KM. Incidence and outcomes of dating violence victimization among high school youth: The role of gender and sexual orientation. *J Interpers Violence*. 2018;33(9):1472-1490.
91. Ehlke SJ, Fitzer SA, Shipley JL, Braitman AL. Exploring the intersection of sexual identity and route of administration in relation to cannabis use among young adult females. *Am J Addict*. 2024;33(3):290-296.
92. Eliason MJ, Burke A, van Olphen J, Howell R. Complex interactions of sexual identity, sex/gender, and religious/spiritual identity on substance use among college students. *Sex Res Social Policy*. 2011;8(2):117-125.
93. Erhabor J, Boakye E, Osuji N, et al. Patterns of tobacco product use and substance misuse among adolescents in the United States. *Prev Med Rep*. 2023;33:102207.
94. Espelage DL, Aragon SR, Birkett M, Koenig BW. Homophobic teasing, psychological outcomes, and sexual orientation among high school students: What influence do parents and schools have? *School Psych Rev*. 2008;37(2):202-216.
95. Estoup AC. Impact of bully victimization and substance use on suicidal behavior in sexual minority youth [dissertation]. Seattle, WA: Seattle Pacific University; 2019.
96. Fallin-Bennett A, Goodin A. Substance use and school characteristics in lesbian, gay, bisexual, and heterosexual high school students. *J Sch Health*. 2019;89(3):219-225.
97. Felt D, Wang X, Ruprecht MM, et al. Differential decline in illicit drug use by sexual identity among United States high school students, 2005–2017. *LGBT Health*. 2020;7(8):420-430.

98. Fernandez J, Gonzalez R, Oves Jr JC, Rodriguez P, Castro G, Barengo NC. Illicit substance use disparities among lesbian, gay, and bisexual high school students in the US in 2017. *J Adolesc Health*. 2021;68(6):1170-1175.
99. Fish JN, Baams L. Trends in alcohol-related disparities between heterosexual and sexual minority youth from 2007 to 2015: Findings from the Youth Risk Behavior Survey. *LGBT Health*. 2018;5(6):359-367.
100. Fish JN, Bishop MD, Russell ST. Developmental differences in sexual orientation and gender identity–related substance use disparities: Findings from population-based data. *J Adolesc Health*. 2021;68(6):1162-1169.
101. Fish JN, Watson RJ, Porta CM, Russell ST, Saewyc EM. Are alcohol-related disparities between sexual minority and heterosexual youth decreasing? *Addiction*. 2017;112(11):1931-1941.
102. Fish JN, Turner B, Phillips G II, Russell ST. Measuring alcohol use across the transition to adulthood: Racial/ethnic, sexual identity, and educational differences. *Addict Behav*. 2018;77:193-202.
103. Fish JN, Watson RJ, Gahagan J, Porta CM, Beaulieu-Prévost D, Russell ST. Smoking behaviours among heterosexual and sexual minority youth? Findings from 15 years of provincially representative data. *Drug Alcohol Rev*. 2018;38(1):101-110.
104. Fish JN, Turner B, Phillips G, Russell ST. Cigarette smoking disparities between sexual minority and heterosexual youth. *Pediatrics*. 2019;143(4):e20181671.
105. Fitzer SA. Psychosocial factors and e-cigarette use: An application of problem behavior theory [master's thesis]. Norfolk, VA: Old Dominion University; 2022.
106. Fonzo M, Cocchio S, Centomo M, et al. Sexual and gender minorities and risk behaviours among university students in Italy. *Int J Environ Res Public Health*. 2021;18(21):11724.
107. Forge N, Hartinger-Saunders R, Wright E, Ruel E. Out of the system and onto the streets: LGBTQ-identified youth experiencing homelessness with past child welfare system involvement. *Child Welfare*. 2018;96(2):47-74.
108. Freibott CE, Stein MD, Lipson SK. The influence of race, sexual orientation and gender identity on mental health, substance use, and academic persistence during the COVID-19 pandemic: A cross-sectional study from a national sample of college students in the healthy minds study. *Drug Alcohol Depend Rep*. 2022;3:100060.
109. Gaiha SM, Lempert LK, Halpern-Felsher B. Underage youth and young adult e-cigarette use and access before and during the coronavirus disease 2019 pandemic. *JAMA Netw Open*. 2020;3(12):e2027572.
110. Gamarel KE, Mereish EH, Colby SM, Barnett NP, Hayes K, Jackson KM. Sexual minority disparities in substance use willingness among youth. *Subst Use Misuse*. 2018;53(1):170-175.
111. Gambadauro P, Carli V, Wasserman D, Balazs J, Sarchiapone M, Hadlaczky G. Serious and persistent suicidality among European sexual minority youth. *PLoS One*. 2020;15(10):e0240840.
112. Garcia LC, Vogel EA, Prochaska JJ. Tobacco product use and susceptibility to use among sexual minority and heterosexual adolescents. *Prev Med*. 2021;145:106384.
113. Gattamorta KA, Salerno JP, Castro AJ. Intersectionality and health behaviors among US high school students: Examining race/ethnicity, sexual identity, and sex. *J Sch Health*. 2019;89(10):800-808.
114. Gattis MN. Psychosocial problems of homeless sexual minority youths and their heterosexual counterparts [dissertation]. St. Louis, MO: Washington University; 2010.
115. Gehring KS, Vaske JC. Out in the open: The consequences of intimate partner violence for victims in same-sex and opposite-sex relationships. *J Interpers Violence*. 2017;32(23):3669-3692.
116. Gerke DR, Atteberry-Ash B, Call J, Hostetter CR. Adolescent substance use at the intersection of sexual orientation and gender identity. *Subst Use Addict J*. 2024;45(4):577–586.

117. Gerke DR, Call J, Atteberry-Ash B, Katz-Kattari S, Kattari L, Hostetter CR. Alcohol use at the intersection of sexual orientation and gender identity in a representative sample of youth in Colorado. *Am J Addict*. 2022;31(1):61-68.
118. Gersh E, Richardson LP, Coker TR, Inwards-Breland DJ, McCarty CA. Same, opposite and both-sex attracted adolescents' mental health, safe-sex practices and substance use. *J Gay Lesbian Ment Health*. 2022;26(2):196-211.
119. Gilbert PA, Kava CM, Afifi R. High-school students rarely use e-cigarettes alone: a sociodemographic analysis of polysubstance use among adolescents in the United States. *Nicotine Tob Res*. 2021;23(3):505-510.
120. Gilmore AK, Leone RM, Oesterle DW, et al. Web-based alcohol and sexual assault prevention program with tailored content based on gender and sexual orientation: preliminary outcomes and usability study of positive change (+ change). *JMIR Form Res*. 2022;6(7):e23823.
121. Glazier RP. Sexual minority youth and risk behaviors: implications for the school environment [dissertation]. Greeley, CO: University of Northern Colorado; 2009.
122. Goodin A, Fallin-Bennett K, Anderson-Hoagland E, Fallin-Bennett A. Tobacco use and mental health disparities in LGB youth. *Public Health Nurs*. 2022;39(3):659-663.
123. Goldbach JT, Mereish EH, Burgess C. Sexual orientation disparities in the use of emerging drugs. *Subst Use Misuse*. 2017;52(2):265-320.
124. Grafsky EL, Letcher A, Slesnick N, Serovich JM. Comparison of treatment response among GLB and non-GLB street-living youth. *Child Youth Serv Rev*. 2011;33(5):569-574.
125. Griffin KW, Lindley LL, Cooper Russell E, Mudd T, Williams C, Botvin GJ. Sexual violence and substance use among first-year university women: Differences by sexual minority status. *Int J Environ Res Public Health*. 2022;19(16):10100.
126. Guimarães NS, de Paula W, de Aguiar AS, Meireles AL. Absence of religious beliefs, unhealthy eating habits, illicit drug abuse, and self-rated health is associated with alcohol and tobacco use among college students—PADu study. *J Public Health*. 2021:1-9.
127. Gumbs CM. Trends in health risk behaviors and victimization among sexual minority youth in Florida: An analysis of youth risk behavior survey data from 2013–2021 [dissertation]. Tallahassee, FL: Florida Agricultural and Mechanical University; 2023.
128. Haardörfer R, Windle M, Fairman RT, Berg CJ. Longitudinal changes in alcohol use and binge-drinking among young-adult college students: Analyses of predictors across system levels. *Addict Behav*. 2021;112:106619.
129. Hahm HC, Wong FY, Huang ZJ, Ozonoff A, Lee J. Substance use among Asian Americans and Pacific Islanders sexual minority adolescents: findings from the National Longitudinal Study of Adolescent Health. *J Adolesc Health*. 2008;42(3):275-283.
130. Hao J, Beld M, Khoddam-Khorasani L, et al. Comparing substance use and mental health among sexual and gender minority and heterosexual cisgender youth experiencing homelessness. *PLoS One*. 2021;16(3):e0248077.
131. Harlow AF, Hendricks PS, Leventhal AM, Barrington-Trimis JL. Psychedelic Microdosing among Young Adults from Southern California. *J Psychoactive Drugs*. 2024:1-12.
132. Harlow AF, Liu F, Young LE, et al. Sexual and Gender Identity Disparities in Nicotine and Tobacco Use Susceptibility and Prevalence: Disaggregating Emerging Identities Among Adolescents From California, USA. *Nicotine Tob Res*. 2024;26(2):203-211.

133. Harlow AF, Lundberg D, Raifman JR, et al. Association of coming out as lesbian, gay, and bisexual+ and risk of cigarette smoking in a nationally representative sample of youth and young adults. *JAMA Pediatr*. 2021;175(1):56-63.
134. Harlow AF, Vogel EA, Tackett AP, et al. Adolescent use of flavored non-tobacco oral nicotine products. *Pediatrics*. 2022;150(3):e2022056586.
135. Hart JL, Ridner SL, Wood LA, et al. Associations between tobacco use patterns and demographic characteristics of sexual minority and heterosexual youth: Results from a nationwide online survey. *Tob Prev Cessat*. 2020;6:69.
136. Hasan A. Tobacco Use Behaviors Among Sexual and Gender Minority Populations [dissertation]. Oklahoma City, OK: The University of Oklahoma Health Sciences Center; 2023.
137. Hatzenbuehler ML. The social environment and suicide attempts in lesbian, gay, and bisexual youth. *Pediatrics*. 2011;127(5):896-903.
138. Hatzenbuehler ML, Corbin WR, Fromme K. Trajectories and determinants of alcohol use among LGB young adults and their heterosexual peers: results from a prospective study. *Dev Psychol*. 2008;44(1):81-90.
139. Hatzenbuehler ML, Jun HJ, Corliss HL, Austin SB. Structural stigma and cigarette smoking in a prospective cohort study of sexual minority and heterosexual youth. *Ann Behav Med*. 2014;47(1):48-56.
140. Hatzenbuehler ML, McLaughlin KA, Xuan Z. Social networks and sexual orientation disparities in tobacco and alcohol use. *J Stud Alcohol Drugs*. 2015;76(1):117-126.
141. Hatzenbuehler ML, Jun HJ, Corliss HL, Austin SB. Structural stigma and sexual orientation disparities in adolescent drug use. *Addict Behav*. 2015;46:14-18.
142. Herchenroeder L, Speed S, Ward RM, Yeung EW. Sexual minority status and food and alcohol disturbance among college students: the moderating role of body esteem. *Eat Behav*. 2023;49:101745.
143. Hinds JT, Loukas A, Perry CL. Sexual and gender minority college students and tobacco use in Texas. *Nicotine Tob Res*. 2018;20(3):383-387.
144. Hinds JT, Loukas A, Perry CL. Explaining sexual minority young adult cigarette smoking disparities. *Psychol Addict Behav*. 2019;33(4):371-381.
145. Hinds JT, Marti CN, Pasch KE, Loukas A. Longitudinal trajectories of marijuana use in tobacco products among young adult Texas college students from 2015 to 2019. *Addiction*. 2023;118(2):372-377.
146. Hirschtritt ME, Dauria EF, Marshall BD, Tolou-Shams M. Sexual minority, justice-involved youth: a hidden population in need of integrated mental health, substance use, and sexual health services. *J Adolesc Health*. 2018;63(4):421-428.
147. Holdren J. An examination of the protective factors that reduce risk behaviors for LGBT high school students in Massachusetts [dissertation]. Santa Barbara, CA: Fielding Graduate University; 2020.
148. Homma Y, Chen W, Poon CS, Saewyc EM. Substance use and sexual orientation among East and Southeast Asian adolescents in Canada. *J Child Adolesc Subst Abuse*. 2012;21(1):32-50.
149. Homma Y, Saewyc E, Zumbo BD. Is it getting better? An analytical method to test trends in health disparities, with tobacco use among sexual minority vs. heterosexual youth as an example. *Int J Equity Health*. 2016;15:79.
150. Hong JS, Valido A, Rivas-Koehl MM, Wade RM, Espelage DL, Voisin DR. Bullying victimization, psychosocial functioning, and protective factors: comparing African American heterosexual and sexual minority adolescents in Chicago's Southside. *J Community Psychol*. 2021;49(5):1358-1375.
151. Horwitz AG, Berona J, Busby DR, et al. Variation in suicide risk among subgroups of sexual and gender minority college students. *Suicide Life Threat Behav*. 2020;50(5):1041-1053.

152. Ibigbami OI, Oginni OA, Bradley C, Lusher J, Sam-Agudu NA, Folayan MO. A cross-sectional study on resilience, anxiety, depression, and psychoactive substance use among heterosexual and sexual minority adolescents in Nigeria. BMC Public Health. 2023;23(1):1759.
153. Ickes M, Hester JW, Wiggins AT, Rayens MK. Juul use among emerging adults transitioning from high school to college. *J Am Coll Health*. 2023;71(1):53-60.
154. Ioerger M, Henry KL, Chen PY, Cigularov KP, Tomazic RG. Beyond same-sex attraction: gender-variant-based victimization is associated with suicidal behavior and substance use for other-sex attracted adolescents. *PLoS One*. 2015;10(9):e0129976.
155. Janssen T, Gamarel KE, Mereish EH, Colby SM, Haikalis M, Jackson KM. Associations among enacted stigma, perceived chances for success, life satisfaction, and substance use among sexual minority and heterosexual youth. *Subst Use Misuse*. 2023;58(9):1121-1131.
156. Jauregui JC, Hong C, Assaf RD, et al. Examining factors associated with cannabis use among sexual and gender minority and cisgender heterosexual emerging adults in California. *LGBT Health*. 2024;11(5):382-391.
157. Jiang Y, Reilly-Chammat R, Cooper T, Viner-Brown S. Disparities in health risk behaviors and health conditions among Rhode Island sexual minority and unsure high school students. *J Sch Health*. 2018;88(11):803-812.
158. Johns MM, Lowry R, Rasberry CN, et al. Violence victimization, substance use, and suicide risk among sexual minority high school students—United States, 2015–2017. *MMWR Morb Mortal Wkly Rep*. 2018;67(43):1211-1215.
159. Johnson SE, O'Brien EK, Coleman B, Tessman GK, Hoffman L, Delahanty, J. Sexual and gender minority U.S. youth tobacco use: Population Assessment of Tobacco and Health (PATH) Study Wave 3, 2015–2016. *Am J Prev Med*. 2019;57(2):256-261.
160. Joung KH, Okoye H, Rana M, Saewyc EM. Trends in substance use among sexual minority adolescents in South Korea. *J Spec Pediatr Nurs*. 2024;29(2):e12425.
161. Jun HJ, Austin SB, Wylie SA, et al. The mediating effect of childhood abuse in sexual orientation disparities in tobacco and alcohol use during adolescence: results from the Nurses' Health Study II. *Cancer Causes Control*. 2010;21(11):1817-1828.
162. Kaczkowski W, Li J, Cooper AC, Robin L. Examining the relationship between LGBTQ-supportive school health policies and practices and psychosocial health outcomes of lesbian, gay, bisexual, and heterosexual students. *LGBT Health*. 2022;9(1):43-53.
163. Kann L, O'Malley Olsen E, McManus T, et al. Sexual identity, sex of sexual contacts, and health-related behaviors among students in grades 9–12—United States and selected sites, 2015. *MMWR Surveill Summ*. 2016;65(9):1-202.
164. Kecojevic A, Wong CF, Schrager SM, et al. Initiation into prescription drug misuse: Differences between lesbian, gay, bisexual, transgender (LGBT) and heterosexual high-risk young adults in Los Angeles and New York. *Addict Behav*. 2012;37(11):1289-1293.
165. Kecojevic A, Jun HJ, Reisner SL, Corliss HL. Concurrent polysubstance use in a longitudinal study of US youth: associations with sexual orientation. *Addiction*. 2017;112(4):614-624.
166. Kelly BC, Weiser JD, Parsons JT. Smoking and attitudes on smoke-free air laws among club-going young adults. *Soc Work Public Health*. 2009;24(5):446-453.
167. Kelly BC, LeClair A, Parsons JT. Methamphetamine use in club subcultures. *Subst Use Misuse*. 2013;48(14):1541-1552.

168. Kelly BC, Wells BE, LeClair A, Tracy D, Parsons JT, Golub SA. Prevalence and correlates of prescription drug misuse among socially active young adults. *Int J Drug Policy*. 2013;24(4):297-303.
169. Kelly BC, Wells BE, Pawson M, LeClair A, Parsons JT. Combinations of prescription drug misuse and illicit drugs among young adults. *Addict Behav*. 2014;39(5):941-944.
170. Kelly BC, Rendina HJ, Vuolo M, Wells BE, Parsons JT. Influences of motivational contexts on prescription drug misuse and related drug problems. *J Subst Abuse Treat*. 2015;48(1):49-55.
171. Kerr DL, Ding K, Chaya J. Substance use of lesbian, gay, bisexual and heterosexual college students. *Am J Health Behav*. 2014;38(6):951-962.
172. Kerr D, Ding K, Burke A, Ott-Walter K. An alcohol, tobacco, and other drug use comparison of lesbian, bisexual, and heterosexual undergraduate women. *Subst Use Misuse*. 2015;50(3):340-349.
173. Khanolkar AR, Frost DM, Tabor E, Redclift V, Amos R, Patalay P. Ethnic and sexual identity-related inequalities in adolescent health and well-being in a national population-based study. *LGBT Health*. 2023;10(1):26-40.
174. Kiekens W, la Roi C, Bos HMW, Kretschmer T, van Bergen DD, Veenstra V. Explaining health disparities between heterosexual and LGB adolescents by integrating the Minority Stress and Psychological Mediation Frameworks: findings from the TRAILS Study. *J Youth Adolesc*. 2020;49:1767-1782.
175. Klare DL, McCabe SE, Ford JA, Schepis TS. Prescription drug misuse, other substance use, and sexual identity: the significance of educational status and psychological distress in US young adults. *Subst Abuse*. 2021;42(3):377-387.
176. Kolto A, Cosma A, Young H, et al. Romantic attraction and substance use in 15-year-old adolescents from eight European countries. *Int J Environ Res Public Health*. 2019;16(17):3063.
177. Kongjareon Y, Samoh N, Peerawaranun P, Guadamuz TE. Pride-based violence, intoxicated sex, and poly-drug use: a vocational school-based study of heterosexual and LGBT students in Bangkok. *BMC Psychiatry*. 2022;22(1):148.
178. Kreski NT, Keyes KM. Disparities in sleep duration among discordant heterosexual adolescents. *J LGBT Youth*. 2022;19(4):429-447.
179. Krueger EA, Barrington-Trimis JL, Unger JB, Leventhal AM. Sexual and gender minority young adult coping disparities during the COVID-19 pandemic. *J Adolesc Health*. 2021;69(5):746-753.
180. Krueger EA, Braymiller JL, Barrington-Trimis JL, Cho J, McConnell RS, Leventhal AM. Sexual minority tobacco use disparities across adolescence and the transition to young adulthood. *Drug Alcohol Depend*. 2020;217:108298.
181. Krueger EA, Hong C, Cunningham NJ, et al. Prevalence of nicotine and tobacco product use by sexual identity, gender identity, and sex assigned at birth among emerging adult tobacco users in California, United States. *Nicotine Tob Res*. 2023;25(7):1378-1385.
182. Krueger EA, Repati ML, Harlow AF, et al. Changes in sexual identity and substance use during young adulthood. *Drug Alcohol Depend*. 2022;241:109674.
183. Kuranz S. Substance use among lesbian, gay, and bisexual young people: the role of neighborhood, school, and family [dissertation]. Boston, MA: Boston University; 2020.
184. Lee J, Tan AS. Intersectionality of sexual orientation with race and ethnicity and associations with E-cigarette use status among US youth. *Am J Prev Med*. 2022;63(5):669-680.
185. Leonard A, Broussard J, Jain J, Kumar S, Santos GM, Dawson-Rose C. Prevalence and correlates of methamphetamine use in transitional age youth experiencing homelessness or housing instability in San Francisco, CA. *J Nurs Scholarsh*. 2023;55(3):711-720.

186. Leone RM, Oesterle D, Yepuri H, et al. College student alcohol use and confidence to intervene in interpersonal violence: Differences by gender and sexual orientation. *J Am Coll Health*. 2024;72(4):1289-1295.
187. Li DH, Turner BC, Mustanski B, Phillips II GL. Sexual orientation disparities in prescription drug misuse among a nationally representative sample of adolescents: prevalence and correlates. *Addict Behav*. 2018;77:143-151.
188. Li J, Haardörfer R, Vu M, Windle M, Berg CJ. Sex and sexual orientation in relation to tobacco use among young adult college students in the US: a cross-sectional study. *BMC Public Health*. 2018;18(1):1244.
189. Li P, Huang Y, Guo L, et al. Sexual attraction and the nonmedical use of opioids and sedative drugs among Chinese adolescents. *Drug Alcohol Depend*. 2018;183:169-175.
190. Liautaud MM, Barrington-Trimis JL, Liu F, et al. E-cigarette, cigarette, and cannabis use patterns as a function of sexual identity in a sample of Southern California young adults. *Addict Behav Rep*. 2021;13:100338.
191. Liu J, Tan AS, Lee J. Vaping of cannabis, cannabidiol, and synthetic cannabis among US sexual minority youths. *JAMA Netw Open*. 2023;6(8):e2329041.
192. London-Nadeau K, Rioux C, Parent S, et al. Longitudinal associations of cannabis, depression, and anxiety in heterosexual and LGB adolescents. *J Abnorm Psychol*. 2021;130(4):333-343.
193. Lorenz TK. Sexual excitation and sex-linked substance use predict overall cannabis use in mostly heterosexual and bisexual women. *Am J Drug Alcohol Abuse*. 2021;47(4):433-443.
194. Lowery S. Health among lesbian, gay, bisexual and questioning teens in Hawai'i: the role of social support in reducing problematic alcohol use and suicide attempts [dissertation]. Honolulu, HI: University of Hawaii at Manoa; 2015.
195. Lowry R, Johns MM, Robin LE, Kann LK. Social stress and substance use disparities by sexual orientation among high school students. *Am J Prev Med*. 2017;53(4):547-558.
196. Lowry R, Kennedy K, Johns MM, Harper CR, Wilkins NJ. Associations between school absence and school violence by sexual identity. *Am J Prev Med*. 2022;63(3):384-391.
197. Loza O, Mangadu T, Ferreira-Pinto JB, Guevara P. Differences in substance use and sexual risk by sexual orientation and gender identity among university and community young adults in a U.S.-Mexico border city. *Health Promot Pract*. 2021;22(4):559-573.
198. Luk JW, Yu J, Haynie DL, Goldstein RB, Simons-Morton BG, Gilman SE. A nationally representative study of sexual orientation and high-risk drinking from adolescence to young adulthood. *J Adolesc Health*. 2023;72(2):222-229.
199. Mantey DS, Yockey RA, Barroso CS. Role of sex on the relationship between sexual minority status and past 30-day marijuana use among high school students (YRBS, 2015–2019). *Addict Behav*. 2021;118:106905.
200. Marshal MP, Sucato G, Stepp SD, et al. Substance use and mental health disparities among sexual minority girls: results from the Pittsburgh Girls Study. *J Pediatr Adolesc Gynecol*. 2012;25(1):15-18.
201. Marshal MP, Burton CM, Chisolm DJ, Sucato GS, Friedman MS. Cross-sectional evidence for a stress-negative affect pathway to substance use among sexual minority girls. *Clin Transl Sci*. 2013;6(4):321-322.
202. Marshall SA, Henry TR, Spivey LA, Rhodes SD, Prinstein MJ, Ip EH. Social context of sexual minority adolescents and relationship to alcohol use. *J Adolesc Health*. 2019;64(5):615-621.
203. Martenson A, Viohl L, Ernst F, Petzold MB, Betzler F. Prevalence and risk factors associated with recreational stimulant use among Berlin college students. *J Subst Use*. 2024;29(4):594-600.

204. Martin-Storey A, Fromme K. Mediating factors explaining the association between sexual minority status and dating violence. *J Interpers Violence*. 2021;36(1-2):132-159.
205. Martin-Storey A, Zhao Z, Toomey RB, Syvertsen AK. Sexual minority identity and risky alcohol use: the moderating role of aggressive behavior. *J Youth Adolesc*. 2024;53(2):1-13.
206. Matthews DD, Blosnich JR, Farmer GW, Adams BJ. Operational definitions of sexual orientation and estimates of adolescent health risk behaviors. *LGBT Health*. 2014;1(1):42-49.
207. Mattingly DT, Agbonlahor O, Hart JL, McLeish AC, Walker KL. Psychological distress and cannabis vaping among US adolescents. *Am J Prev Med*. 2024;66(3):534-539.
208. McCabe CJ. Examining stress-related pathways of substance use among sexual minority women [dissertation]. Seattle, WA: University of Washington; 2019.
209. McCabe CJ, Hipwell AE, Keenan K, Stepp SD, Chung T, King KM. Substance use and sexual-minority status: examining the mediating roles of stress and emotion dysregulation in young adult women. *Clin Psychol Sci*. 2021;9(6):1095-1114.
210. McCabe CJ, Rhew IC, Walukevich-Dienst K, Graupensperger S, Lee CM. Increased coping motives during the COVID-19 pandemic widen cannabis disparities between sexual minoritized and non-minoritized young adults: a bimonthly assessment of data preceding and spanning the pandemic. *Psychol Addict Behav*. 2023;37(5):670-680.
211. McCabe SE, Hughes TL, Bostwick W, Morales M, Boyd CJ. Measurement of sexual identity in surveys: implications for substance abuse research. *Arch Sex Behav*. 2012;41(3):649-657.
212. McCurdy AL, Gower AL, Rider GN, et al. Adolescent substance use at the intersections of foster care, sexual orientation and gender identity, racial/ethnic identity, and sex assigned at birth. *Child Abuse Negl*. 2023;137:106042.
213. McGraw LK, Tyler KA, Simons LG. Risk factors for sexual assault of heterosexual and sexual minority college women. *J Interpers Violence*. 2022;37(9-10):NP8032-NP8055.
214. McLaughlin KA, Hatzenbuehler ML, Xuan Z, Conron KJ. Disproportionate exposure to early-life adversity and sexual orientation disparities in psychiatric morbidity. *Child Abuse Negl*. 2012;36(9):645-655.
215. Mereish EH, Goldbach JT, Burgess C, DiBello AM. Sexual orientation, minority stress, social norms, and substance use among racially diverse adolescents. *Drug Alcohol Depend*. 2017;178:49-56.
216. Mereish EH, Sheskier M, Hawthorne DJ, Goldbach JT. Sexual orientation disparities in mental health and substance use among Black American young people in the USA: effects of cyber and bias-based victimization. *Cult Health Sex*. 2019;21(9):985-998.
217. Michael SS. Smoking in a sexual minority population: a comparison of two adolescent cohorts [dissertation]. Tucson, AZ: University of Arizona; 2016.
218. Mitchell KJ, Ybarra ML, Goodman KL, Strøm IF. Polyvictimization among sexual and gender minority youth. *Am J Prev Med*. 2023;65(2):182-191.
219. Morgan PD. Bullying and substance use among American Indian/Alaskan Native youth: secondary data analysis of 2011–2019 Youth Risk Behavior Surveillance Data [dissertation]. Philadelphia, PA: Temple University; 2021.
220. Muster RL. Resilience as a predictor of non-medicinal use of prescription drugs among college students [dissertation]. Kent, OH: Kent State University; 2021.
221. Nguyen N, McQuoid J, Neilands TB, et al. Same-day use of cigarettes, alcohol, and cannabis among sexual minority and heterosexual young adult smokers. *Psychol Addict Behav*. 2021;35(2):215-223.
222. Nieves AC. Bullying, sexual identity, health risk behaviors and outcomes among adolescent youth in a metropolitan school district [dissertation]. Fort Lauderdale, FL: Nova Southeastern University; 2017.

223. Noel JK, Tudela SE, Rosenthal SR. Sexual minority status, illicit drug use, and depressive symptoms. *J LGBT Youth*. 2024;21(3):490-506.
224. Oh HY, Jacob L, Smith L, et al. Sexual minority status and psychotic experiences among young adult college students in the United States. *J Homosex*. 2024;71(4):916-933.
225. Ortiz-Hernandez L, Tello BLG, Valdes J. The association of sexual orientation with self-rated health, and cigarette and alcohol use in Mexican adolescents and young adults. *Soc Sci Med*. 2009;69(1):85-93.
226. Ott MQ, Clark MA, Balestrieri SG, Gamarel KE, Barnett NP. Social networks and sexual and gender minority disparities in alcohol use and consequences among first-year college students. *LGBT Health*. 2022;9(7):489-495.
227. Ott MQ, Wypij D, Corliss HL, et al. Repeated changes in reported sexual orientation identity linked to substance use behaviors in youth. *J Adolesc Health*. 2013;52(4):465-472.
228. Pachankis JE, Westmaas JL, Dougherty LR. The influence of sexual orientation and masculinity on young men’s tobacco smoking. *J Consult Clin Psychol*. 2011;79(2):142-152.
229. Pereira FA, de Oliveira LG, da Silva GT, Scatena A, Kim HS, Andrade ALM. Validation of Alcohol Use Disorders Identification Test (AUDIT) in Brazilian colleges: network analysis, measurement invariance, and screening efficiency. *Int J Ment Health Addict*. 2024;1-19.
230. Pesola F, Shelton KH, van den Bree MBM. Sexual orientation and alcohol problem use among UK adolescents: an indirect link through depressed mood. *Addiction*. 2014;109(7):1072-1080.
231. Phillips II G, Turner B, Felt D, Han Y, Marro R, Beach LB. Trends in alcohol use behaviors by sexual identity and behavior among high school students, 2007–2017. *J Adolesc Health*. 2019;65(6):760-768.
232. Phillips II G, Wang X, Ruprecht MM, et al. Differential alcohol use disparities by sexual identity and behavior among high school students. *Alcohol Alcohol*. 2021;56(4):490-499.
233. Pigeon B. The effects of legalized marijuana on teens [dissertation]. Denver, CO: University of Colorado at Denver; 2020.
234. Pollard MS, Tucker JS, Green HD, Kennedy DP, Go MH. Romantic attraction and adolescent smoking trajectories. *Addict Behav*. 2011;36(12):1275-1281.
235. Poteat V, Aragon SR, Espelage DL, Koenig BW. Psychosocial concerns of sexual minority youth: complexity and caution in group differences. *J Consult Clin Psychol*. 2009;77(1):196-201.
236. Price-Feeney M, Ybarra ML, Mitchell KJ. Health indicators of lesbian, gay, bisexual, and other sexual minority youth living in rural communities. *J Pediatr*. 2019;205:236-243.
237. Prud’homme J, Hofer MK, Ames ME, Turner BJ. Disparities in the prevalence, frequency, and trajectories of substance use and disordered eating across first-year university in sexual minority undergraduates. *J Am Coll Health*. 2022;1-13.
238. Qeadan F, Egbert J, Barbeau WA, Madden EF, Venner KL, English K. Sexuality and gender identity inequities in substance use disorder and its treatment among American Indian, Alaska Native, and Native Hawaiian college students. *Subst Use Misuse*. 2022;57(14):2085-2093.
239. Ragavan MI, Culyba AJ, Randell KA, Miller E, Chu KH. Electronic vapor product use and violence victimization among a nationally representative sample of adolescents. *J Adolesc Health*. 2021;68(2):422-425.
240. Ramo DE, Grov C, Delucchi K, Kelly BC, Parsons JT. Typology of club drug use among young adults recruited using time-space sampling. *Drug Alcohol Depend*. 2010;107(2-3):119-127.
241. Ranker LR, Lipson SK. Prevalence of heavy episodic drinking and alcohol use disorder diagnosis among US college students: results from the national Healthy Minds Study. *Addict Behav*. 2022;135:107452.

242. Ray CM, Tyler KA, Gordon Simons L. Risk factors for forced, incapacitated, and coercive sexual victimization among sexual minority and heterosexual male and female college students. *J Interpers Violence*. 2021;36(5-6):2241-2261.
243. Remafedi G, Jurek AM, Oakes JM. Sexual identity and tobacco use in a venue-based sample of adolescents and young adults. *Am J Prev Med*. 2008;35(6 Suppl 1):S463-S470.
244. Rentería R, Benjet C, Gutiérrez-García RA, et al. Prevalence of 12-month mental and substance use disorders in sexual minority college students in Mexico. *Soc Psychiatry Psychiatr Epidemiol*. 2021;56(2):247-257.
245. Rocheleau GC, Rocheleau BN, Piatak KA, Thompson IJ. LGBTQ+ identity and delinquency: the importance of negative emotions and social bonds. *Crim Justice Stud*. 2024;1-21.
246. Romm KF, Berg CJ. Disparities in transitions from e-cigarette use to other tobacco use patterns among sexual minority versus heterosexual women and men in the United States. *Subst Use Addict J*. 2024;45(2):297-327.
247. Romm KF, Berg CJ. Patterns of adverse childhood experiences and problematic health outcomes among US young adults: a latent class analysis. *Subst Use Addict J*. 2024;45(2):191-200.
248. Romm KF, Cohn AM, Beebe LA, Berg CJ. Disparities in cannabis use outcomes, perceived risks, and social norms across sexual orientation groups of US young adult women and men. *Health Educ Res*. 2023;38(6):513-526.
249. Romm KF, Cohn AM, Wang Y, Berg CJ. Psychosocial predictors of trajectories of dual cigarette and e-cigarette use among young adults in the US. *Addict Behav*. 2023;141:107658.
250. Romm KF, Huebner DM, Pratt-Chapman ML, et al. Disparities in traditional and alternative tobacco product use across sexual orientation groups of young adult men and women in the US. *Subst Abuse*. 2022;43(1):815-824.
251. Romm KF, Patterson B, Arem H, Price OA, Wang Y, Berg CJ. Cross-sectional retrospective assessments versus longitudinal prospective assessments of substance use change among young adults during COVID-19: magnitude and correlates of discordant findings. *Subst Use Misuse*. 2022;57(3):484-489.
252. Rosario M, Corliss HL, Everett BG, et al. Sexual orientation disparities in cancer-related risk behaviors of tobacco, alcohol, sexual behaviors, and diet and physical activity: pooled youth risk behavior surveys. *Am J Public Health*. 2014;104(2):245-254.
253. Rosario M, Reisner SL, Corliss HL, Wypij D, Calzo J, Austin SB. Sexual-orientation disparities in substance use in emerging adults: a function of stress and attachment paradigms. *Psychol Addict Behav*. 2014;28(3):790-804.
254. Rosario M, Li F, Wypij D, et al. Disparities by sexual orientation in frequent engagement in cancer-related risk behaviors: a 12-year follow-up. *Am J Public Health*. 2016;106(4):698-706.
255. Russell ST, McCurdy AL. Examination of the “model minority” stereotype through ethnicity and sexual orientation heterogeneity among Asian American youth. *J Adolesc*. 2023;95(6):1258-1273.
256. Saewyc E, Clark T, Barney L, Brunanski D, Homma Y. Enacted stigma and HIV risk behaviors among sexual minority Indigenous youth in Canada, New Zealand, and the United States. *Pimatisiwin*. 2014;11(3):411-420.
257. Santa Maria DM, Narendorf SC, Cross MB. Prevalence and correlates of substance use in homeless youth and young adults. *J Addict Nurs*. 2018;29(1):23-31.
258. Saw YM, Saw TN, Yasuoka J, et al. Gender difference in early initiation of methamphetamine use among current methamphetamine users in Muse, Northern Shan State, Myanmar. *Harm Reduct J*. 2017;14(1):21.

259. Scannapieco M, Painter KR, Blau G. A comparison of LGBTQ youth and heterosexual youth in the child welfare system: mental health and substance abuse occurrence and outcomes. *Child Youth Serv Rev*. 2018;91:39-46.
260. Schauer GL, Berg CJ, Bryant LO. Sex differences in psychosocial correlates of concurrent substance use among heterosexual, homosexual, and bisexual college students. *Am J Drug Alcohol Abuse*. 2013;39(4):252-258.
261. Scheer JR, Antebi-Gruszka N, Sullivan T. Physical and sexual victimization class membership and alcohol misuse and consequences among sexual minority and heterosexual female youth. *Psychol Violence*. 2021;11(5):434-444.
262. Scheer JR, McConocha E, Behari K, Pachankis JE. Sexual violence as a mediator of sexual orientation disparities in alcohol use, suicidality, and sexual-risk behavior among female youth. *Psychol Sex*. 2021;12(1-2):37-51.
263. Schipani-McLaughlin AM, Nielsen KE, Mosley EA, et al. Alcohol use and alcohol-related consequences based on gender and sexual orientation among college students. *Am J Addict*. 2022;31(3):189-199.
264. Schroeder GE, McMillan IF, Jules BN, Langhinrichsen-Rohling J. Bad break-up behavior: break-up characteristics, emotional dysregulation, and alcohol use as risk factors for cyber dating abuse among sexual orientation diverse college students. *J Interpers Violence*. 2024;39(13-14):3135-3157.
265. Schuler MS, Rice CE, Evans-Polce RJ, Collins RL. Disparities in substance use behaviors and disorders among adult sexual minorities by age, gender, and sexual identity. *Drug Alcohol Depend*. 2018;189:139-146.
266. Schuler MS, Stein BD, Collins RL. Differences in substance use disparities across age groups in a national cross-sectional survey of lesbian, gay, and bisexual adults. *LGBT Health*. 2019;6(2):68-76.
267. Schwartz SE, Ross SG, Bryant JA, Duncan JD. Mental and physical health among students at a private university that held in-person classes during the COVID-19 pandemic. *J Am Coll Health*. 2024;72(2):587-597.
268. Seil KS, Desai MM, Smith MV. Sexual orientation, adult connectedness, substance use, and mental health outcomes among adolescents: findings from the 2009 New York City Youth Risk Behavior Survey. *Am J Public Health*. 2014;104(10):1950-1956.
269. Selby VL. Synthetic cannabinoid use and clinical correlates among youth in treatment for substance use disorders [dissertation]. Baltimore, MD: University of Maryland; 2017.
270. Shadick R, Dagirmanjian FB, Trub L, Dawson H. Sexual orientation and first-year college students' nonmedical use of prescription drugs. *J Am Coll Health*. 2016;64(4):292-299.
271. Shechory-Bitton M, Bonny-Noach H. Psychological factors and the use of psychoactive substances in relation to sexual orientation: A study on Israeli young adults. *Curr Psychol*. 2023;42(24):20452-20462.
272. Shpiegel S, Sussman S, Sherman SE, El Shahawy O. Smoking behaviors among adolescents in foster care: a gender-based analysis. *Subst Use Misuse*. 2017;52(11):1469-1477.
273. Shyhalla K, Smith DM, Diaz A, et al. Changes in cannabis, tobacco, and alcohol use among sexually active female adolescents and young adults over a twelve-year period ending in 2019. *Addict Behav*. 2021;121:106994.
274. Siconolfi D, Davis JP, Pedersen ER, et al. Trajectories of emerging adults' binge drinking and depressive symptoms and associations with sexual violence victimization: examining differences by sexual and gender minority status. *J Interpers Violence*. 2023;38(7-8):6085-6112.

275. Silveira ML, Green VR, Iannaccone R, Kimmel HL, Conway KP. Patterns and correlates of polysubstance use among US youth aged 15–17 years: Wave 1 of the Population Assessment of Tobacco and Health (PATH) Study. *Addiction*. 2019;114(5):907-916.
276. Singer ER. Resilience among sexual minority youth: the role of natural mentors in improving mental health and substance abuse outcomes [dissertation]. Chestnut Hill, MA: Boston College; 2015.
277. Sironi M, Fricke J. Sexual orientation and health behaviors among college students in Italy. *Genus*. 2021;77(1):26.
278. Skye M, Craig S, Donald C, et al. Are American Indian/Alaska Native adolescent health behaviors different? A review of AI/AN youth involved in native STAND curriculum, 2014–2017 United States. *Matern Child Health J*. 2021;25:1893-1902.
279. Spinardi-Pirozzi J. A comparison of drinking behavior between heterosexual and gay, lesbian, and bisexual college students: an examination of prevalence and contributing factors [dissertation]. New York, NY: Pace University; 2009.
280. Stogner J, Miller BL. Exploring the relationships between sexual orientation and gender identity and youth synthetic cannabinoid use. *Subst Use Misuse*. 2020;56(2):327-332.
281. Stogner J, Patterson C. Suicidal ideation, planning, and attempts among synthetic cannabinoid users across different demographic subgroups. *Crisis*. 2022;43(4):323-330.
282. Suárez DE, Cardozo AC, Villarreal ME, Trujillo EM. Non-heterosexual medical students are critically vulnerable to mental health risks: the need to account for sexual diversity in wellness initiatives. *Teach Learn Med*. 2020;33(1):1-9.
283. Tabaac AR, Charlton BM, Tan ASL, Cobb CO, Sutter ME. Differences in tobacco product use by sexual orientation and violence factors among United States youth. *J Pediatr*. 2021;233:241-248.
284. Talley AE, Grimaldo G, Wilsnack SC, Hughes TL, Kristjanson AF. Childhood victimization, internalizing symptoms, and substance use among women who identify as mostly heterosexual. *LGBT Health*. 2016;3(4):266-274.
285. Talley AE, Turner B, Foster AM, Phillips G. Sexual minority youth at risk of early and persistent alcohol, tobacco, and marijuana use. *Arch Sex Behav*. 2019;48(4):1073-1086.
286. Thurston IB, Dietrich J, Bogart LM, et al. Correlates of sexual risk among sexual minority and heterosexual South African youth. *Am J Public Health*. 2014;104(7):1265-1269.
287. Travers Á, Armour C, Hansen M, et al. Lesbian, gay, or bisexual identity as a risk factor for trauma and mental health problems in Northern Irish students and the protective role of social support. *Eur J Psychotraumatol*. 2020;11(1):1708144.
288. Tucker JS, Ellickson PL, Klein DJ. Understanding differences in substance use among bisexual and heterosexual young women. *Womens Health Issues*. 2008;18(5):387-398.
289. Tyler KA, Schmitz RM. Bullying at school and on the street: risk factors and outcomes among homeless youth. *J Interpers Violence*. 2018;33(14):2237-2257.
290. Veldhuis CB, George M, Everett BG, Liu J, Hughes TL, Bruzzese JM. The association of asthma, sexual identity, and inhaled substance use among US adolescents. *Ann Am Thorac Soc*. 2021;18(2):273-280.
291. Veliz P, Boyd CJ, McCabe SE. Substance use among adolescent sexual minority athletes: a secondary analysis of the youth risk behavior survey. *Addict Behav Rep*. 2016;4:18-23.
292. Veliz P, Epstein-Ngo Q, Zdroik J, Boyd CJ, McCabe SE. Substance use among sexual minority collegiate athletes: a national study. *Subst Use Misuse*. 2016;51(4):517-532.
293. Vock F, Johnson-Ferguson L, Bechtiger L, et al. Substance use in sexual minority youth: prevalence in an urban cohort. *Child Adolesc Psychiatry Ment Health*. 2023;17(1):109.

294. Walukevich-Dienst K, Twitty TD, Buckner JD. Sexual minority women and cannabis use: the serial impact of PTSD symptom severity and coping motives. *Addict Behav*. 2019;92:1-5.
295. Wang Y, Duan Z, Romm KF, et al. Bidirectional associations between depressive symptoms and cigarette, e-cigarette, cannabis, and alcohol use: cross-lagged panel analyses among young adults before and during COVID-19. *Addict Behav*. 2022;134:107422.
296. Wang Y, Xu S, Zhang X, et al. Effects of tobacco versus electronic cigarette usage on nonsuicidal self-injury and suicidality among Chinese youth: cross-sectional self-report survey study. *JMIR Public Health Surveill*. 2023;9:e47058.
297. Wedel AV, Goodhines PA, Zaso MJ, Park A. Prospective associations of discrimination, race, and sexual orientation with substance use in adolescents. *Subst Use Misuse*. 2022;57(2):263-272.
298. White Hughto JM, Biello KB, Reisner SL, Perez-Brumer A, Heflin KJ, Mimiaga MJ. Health risk behaviors in a representative sample of bisexual and heterosexual female high school students in Massachusetts. *J Sch Health*. 2016;86(1):61-71.
299. Wichaidit W, Mattawanon N, Somboonmark W, Prodtongsom N, Chongsuvivatwong V, Assanangkornchai S. Behavioral health and experience of violence among cisgender heterosexual and LGBTQ+ adolescents in Thailand. *PLoS One*. 2023;18(6):e0287130.
300. Wilson JD, Sumetsky NM, Coulter RWS, Liebschutz J, Miller E, Mair CF. Opioid-related disparities in sexual minority youth, 2017. *J Addict Med*. 2020;14(6):475-479.
301. Wishart M, Davis C, Pavlis A, Hallam KT. Increased mental health and psychosocial risks in LGBQ youth accessing Australian youth AOD services. *J LGBT Youth*. 2020;17(3):331-349.
302. Woodford MR, Krentzman AR, Gattis MN. Alcohol and drug use among sexual minority college students and their heterosexual counterparts: the effects of experiencing and witnessing incivility and hostility on campus. *Subst Abuse Rehabil*. 2012;3:11-23.
303. Zhang H, Wong WCW, Ip P, Fan S, Yip PSF. Health status and risk behaviors of sexual minorities among Chinese adolescents: a school-based survey. *J Homosex*. 2017;64(3):382-396.
304. Zhang L, Finan LJ, Bersamin M, Fisher DA, Paschall MJ. Sexual orientation–based alcohol, tobacco, and other drug use disparities: the protective role of school-based C centers. *Youth Soc*. 2020;52(7):1153-1173.

Table S1. Meta-regression of effect sizes on standard errors using Robust Variance Estimation

Substance outcome types	n (k)	Hedges' g	Standard Error	95% CI		p-value	τ^2	I^2 (%)	Q-statistic	Q-statistic (p-value)
**Continuous outcome**										
All substance outcomes	360 (70)	1.24	0.46	0.16	2.33	0.03	0.23	100.00	200,000	<0.001
**Substance outcome types**	**n (k)**	**Odds Ratio**	**Standard Error**	**95% CI**		**p-value**	τ^2	I^2 **(%)**	**Q-statistic**	**Q-statistic (p-value)**
**Dichotomous outcome**										
All substance outcomes	4311 (232)	1.24	0.10	1.02	1.50	0.03	0.24	98.32	4694.56	<0.001

Abbreviations: CI, confidence interval; k, number of studies; n, number of estimates; SD, standardized deviation. We calculated Hedges' g to measure standardized mean differences with small study bias correction in substance use between SMY and heterosexual youth.

Note. This table display only studies reporting quantity and frequency outcomes for substance use. Studies measuring other outcome types (i.e., age of initiation) were excluded due to small sample sizes.

Figure S1. Number of included studies by publication year (k = 304)

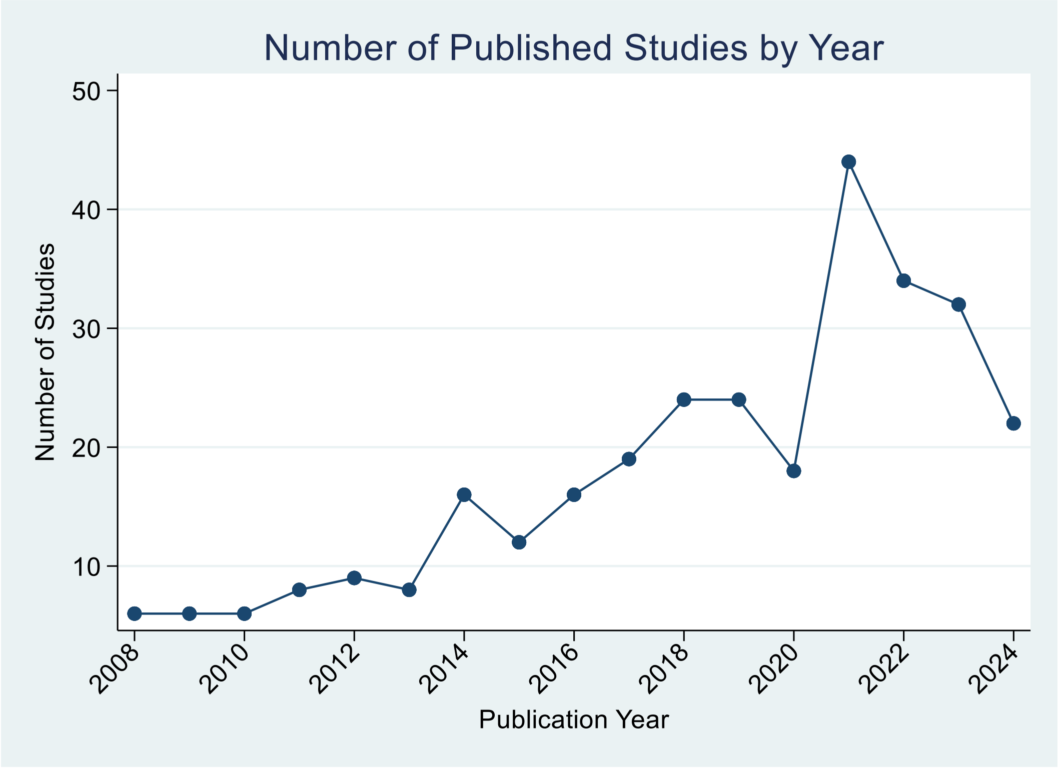

Figure S2. Contour-enhanced funnel plot for continuous outcomes

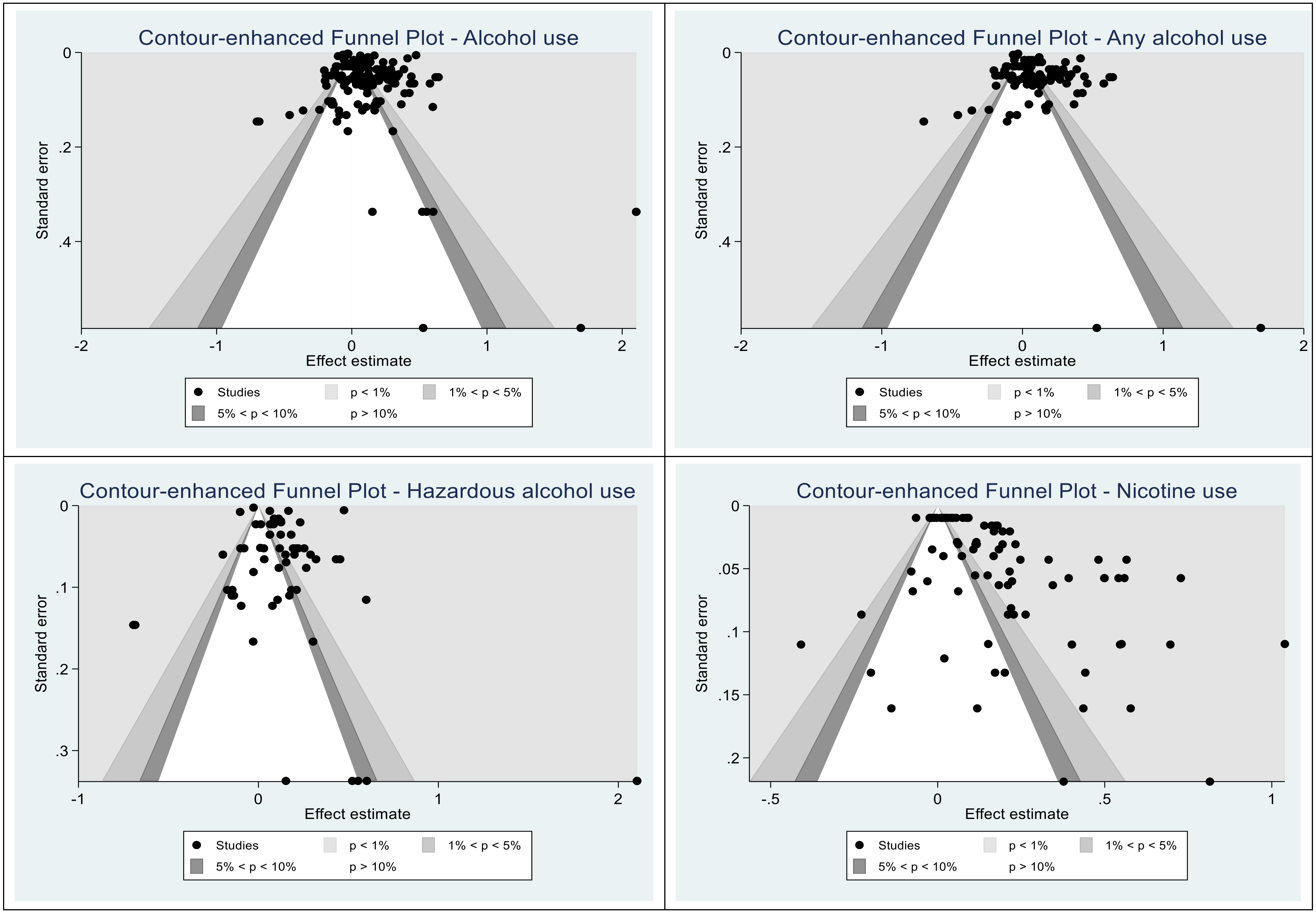

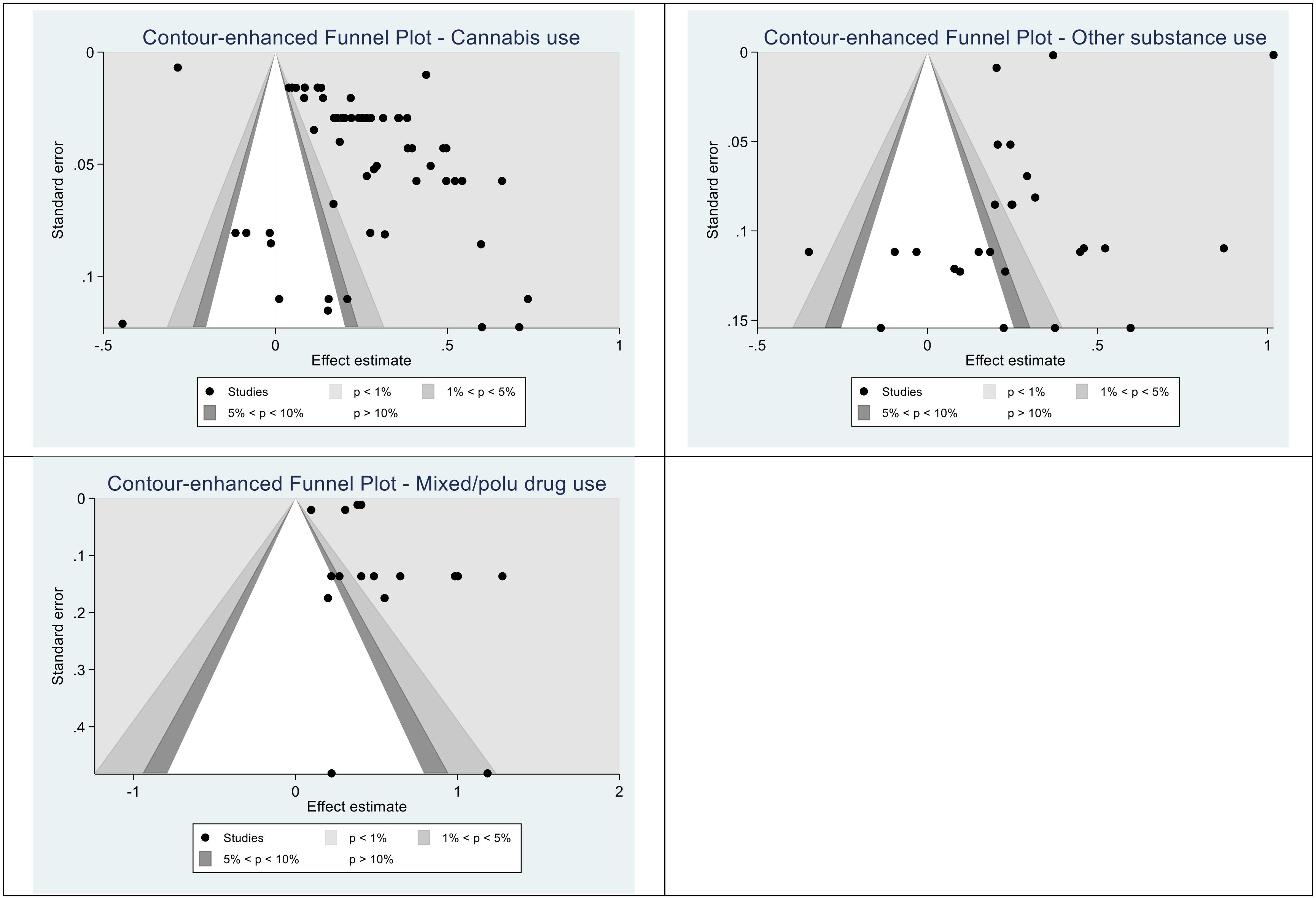

Note. Contour-enhanced funnel plots display only studies reporting quantity and frequency outcomes for substance use. Studies measuring other outcome types (i.e., age of initiation) were excluded from these visualizations.

Supplemental Online Content

Figure S3. Contour-enhanced funnel plot for dichotomous outcomes

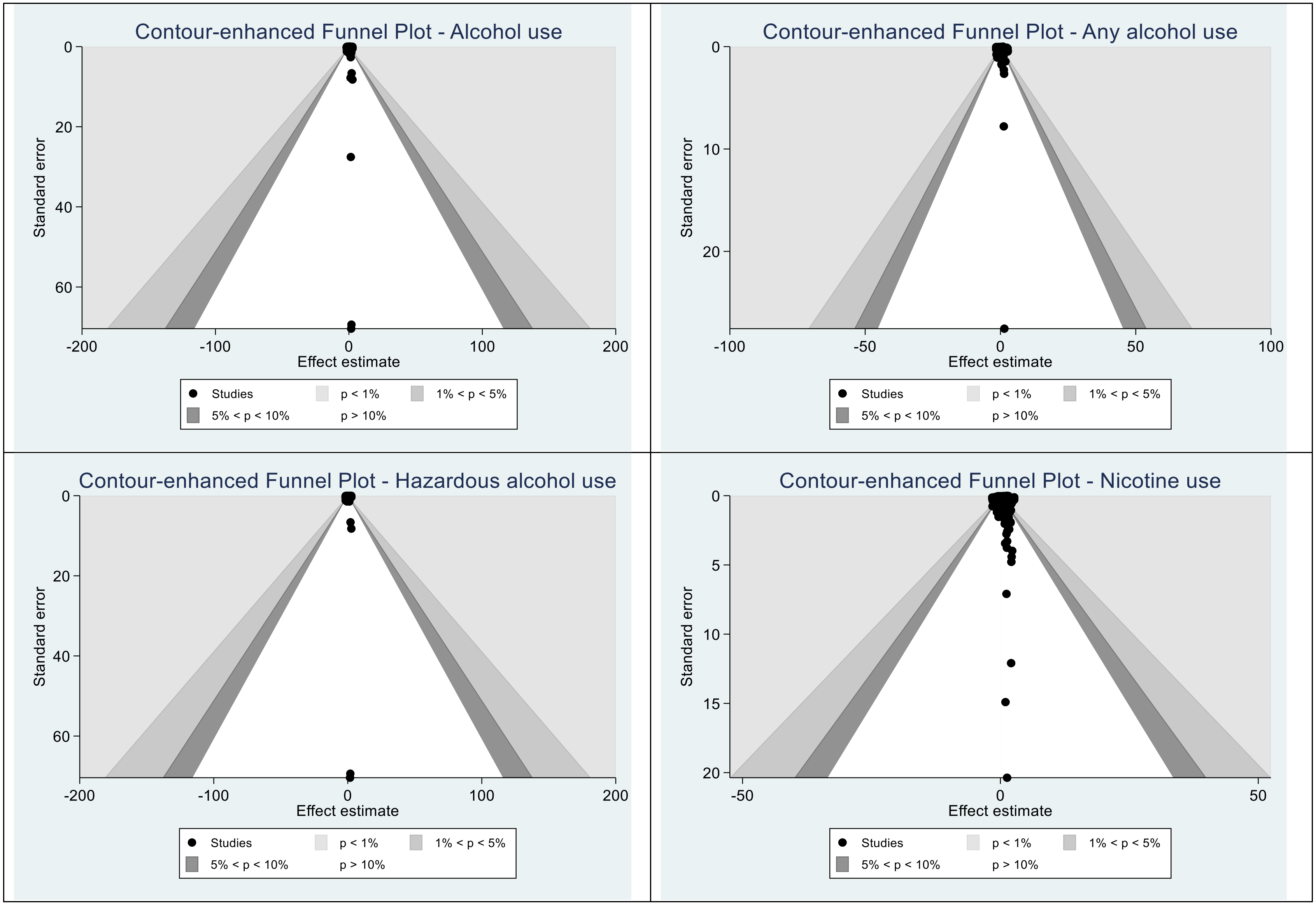

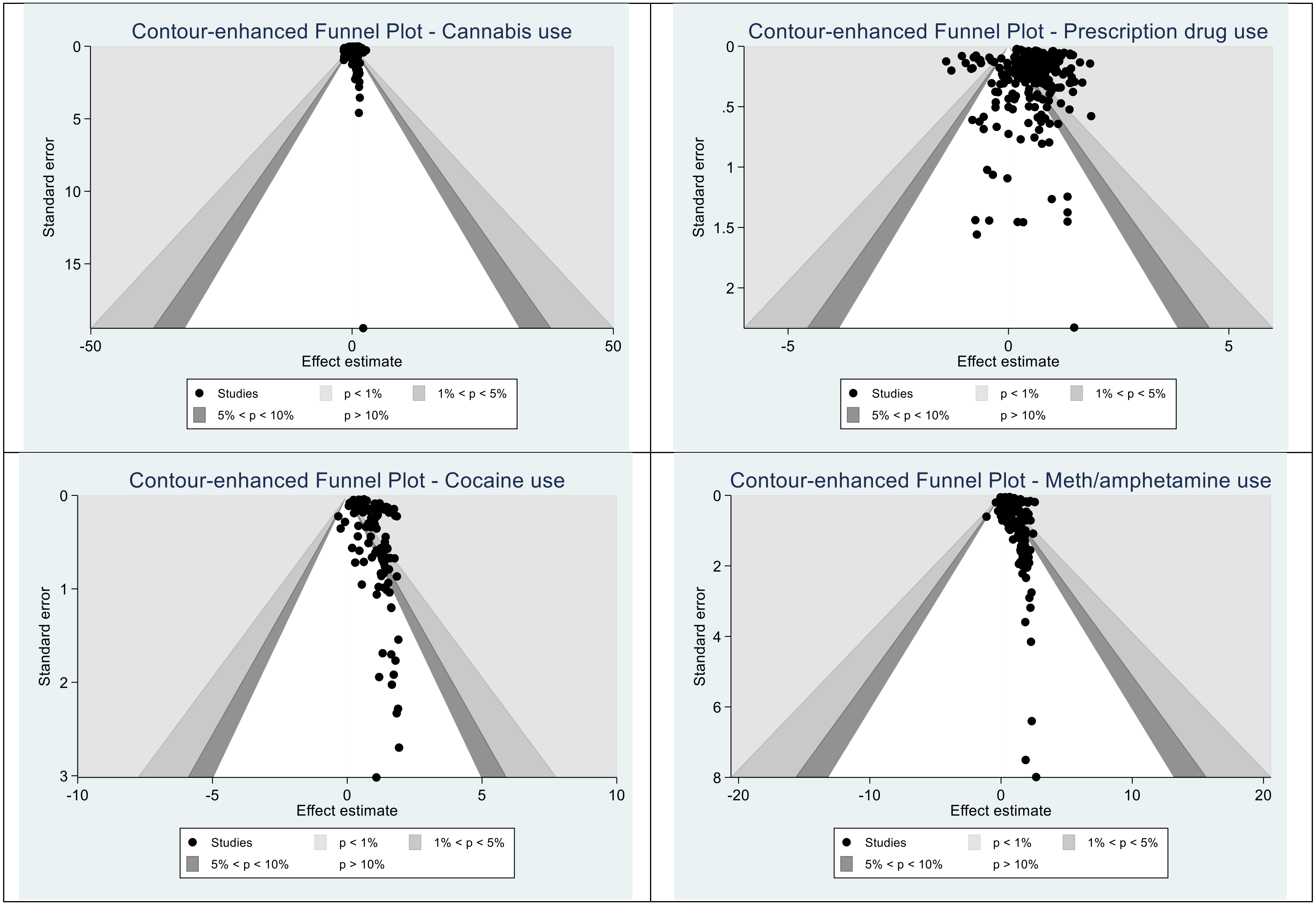
Contour-enhanced Funnel Plot - Cannabis use
Standard error
Effect estimate
0
5
10
15
-50
0
50
Studies
p < 1%
1% < p < 5%
5% < p < 10%
p > 10%
Contour-enhanced Funnel Plot - Prescription drug use
Standard error
Effect estimate
0
.5
1
1.5
2
-5
0
5
Studies
p < 1%
1% < p < 5%
5% < p < 10%
p > 10%
Contour-enhanced Funnel Plot - Cocaine use
Standard error
Effect estimate
0
1
2
3
-10
-5
0
5
10
Studies
p < 1%
1% < p < 5%
5% < p < 10%
p > 10%
Contour-enhanced Funnel Plot - Meth/amphetamine use
Standard error
Effect estimate
0
2
4
6
8
-20
-10
0
10
20
Studies
p < 1%
1% < p < 5%
5% < p < 10%
p > 10%

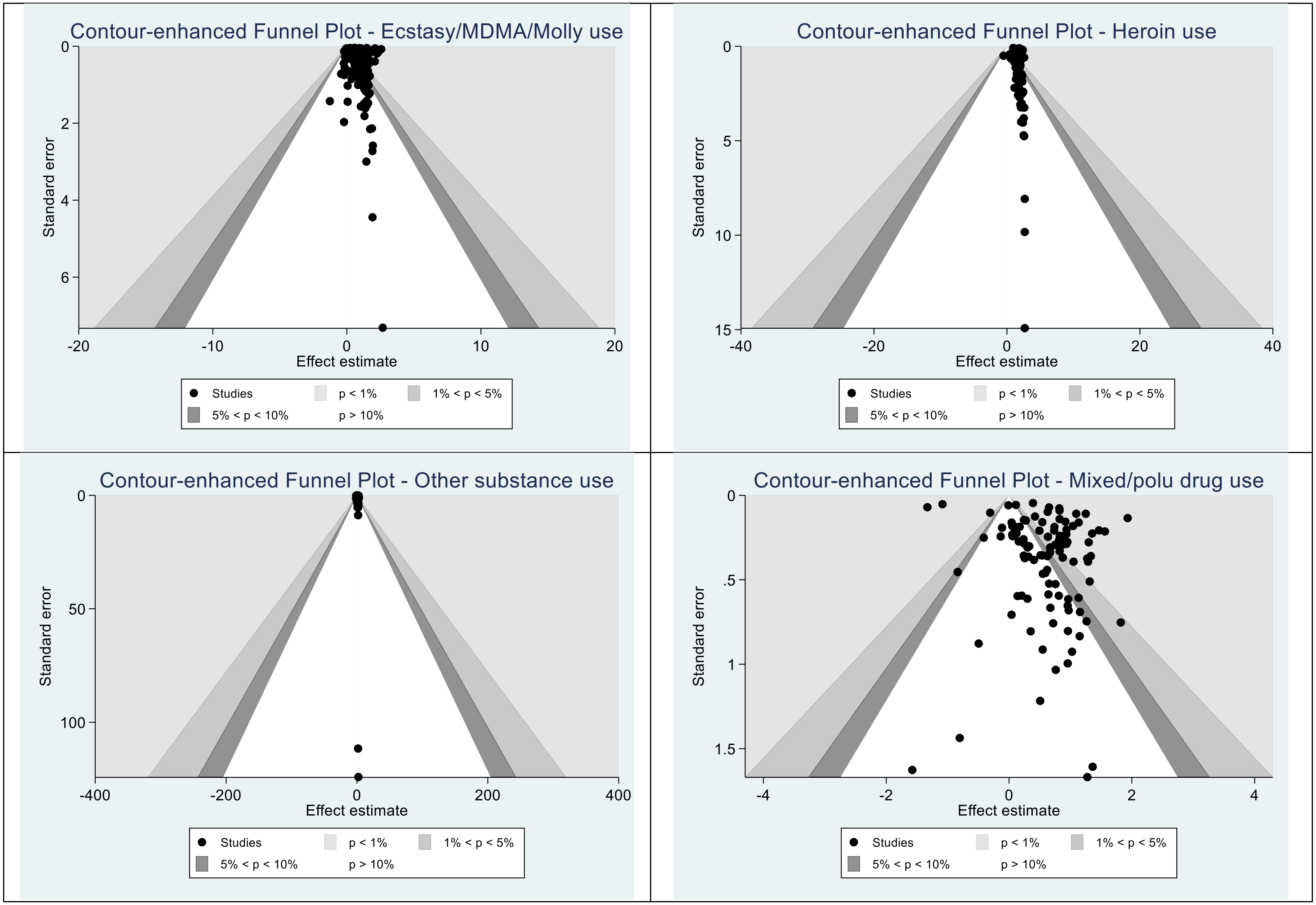

Note. Contour-enhanced funnel plots display only studies reporting quantity and frequency outcomes for substance use. Studies measuring other outcome types (i.e., age of initiation) were excluded from these visualizations.
